# Multi-Granularity Whole-Brain Segmentation Based Functional Network Analysis Using Resting-State fMRI

**DOI:** 10.3389/fnins.2018.00942

**Published:** 2018-12-12

**Authors:** Yujing Gong, Huijun Wu, Jingyuan Li, Nizhuan Wang, Hanjun Liu, Xiaoying Tang

**Affiliations:** ^1^Department of Electrical and Electronic Engineering, Southern University of Science and Technology, Shenzhen, China; ^2^School of Biomedical Engineering, Health Science Center, Shenzhen University, Shenzhen, China; ^3^Department of Electrical and Computer Engineering, Carnegie Mellon University, Pittsburgh, PA, United States; ^4^Department of Rehabilitation Medicine, The First Affiliated Hospital, Sun Yat-sen University, Guangzhou, China; ^5^Guangdong Provincial Key Laboratory of Brain Function and Disease, Zhongshan School of Medicine, Sun Yat-sen University, Guangzhou, China

**Keywords:** multi-granularity, ontology relationship, resting-state, fMRI, brain network, small-worldness, multi-atlas segmentation

## Abstract

In this work, we systematically analyzed the effects of various nodal definitions, as determined by a multi-granularity whole-brain segmentation scheme, upon the topological architecture of the human brain functional network using the resting-state functional magnetic resonance imaging data of 19 healthy, young subjects. A number of functional networks were created with their nodes defined according to two types of anatomical definitions (Type I and Type II) each of which consists of five granularity levels of whole brain segmentations with each level linked through ontology-based, hierarchical, structural relationships. Topological properties were computed for each network and then compared across levels within the same segmentation type as well as between Type I and Type II. Certain network architecture patterns were observed in our study: (1) As the granularity changes, the absolute values of each node's nodal degree and nodal betweenness change accordingly but the relative values within a single network do not change considerably; (2) The average nodal degree is generally affected by the sparsity level of the network whereas the other topological properties are more specifically affected by the nodal definitions; (3) Within the same ontology relationship type, as the granularity decreases, the network becomes more efficient at information propagation; (4) The small-worldness that we observe is an intrinsic property of the brain's resting-state functional network, independent of the ontology type and the granularity level. Furthermore, we validated the aforementioned conclusions and measured the reproducibility of this multi-granularity network analysis pipeline using another dataset of 49 healthy young subjects that had been scanned twice.

## Introduction

It has been suggested that one may view the human brain as a complex yet highly efficient network that is composed of multiple anatomical regions (van den Heuvel and Hulshoff Pol, 2010). The advent of advanced neuroimaging techniques has enabled active research on such brain networks, largely centered around three primary types, the structural network analyzed via the tractography of diffusion tensor imaging (DTI) (Hagmann et al., 2007), the morphological network analyzed via structural magnetic resonance imaging (MRI) (He et al., 2007), as well as the functional network analyzed via electroencephalography (EEG) (Mantini et al., 2007), magnetoencephalography (MEG) (Stam, 2004), or functional MRI (fMRI) (Achard et al., 2006). Recently, resting-state fMRI (rs-fMRI) based functional network analysis has gained a large amount of research attention for its unveiling of the topological patterns of human brain networks “at rest” (De Luca et al., 2006; Greicius et al., 2009; van den Heuvel and Hulshoff Pol, 2010).

In brain network analysis, graph theory is a widely employed technique that decomposes a network into a set of nodes and between-node edges. In a brain network, the nodes usually refer to different anatomical regions. Most rs-fMRI studies have utilized the 90 cortical regions as defined in the Automated Anatomical Labeling (AAL) atlas (Tzourio-Mazoyer et al., 2002) to serve as nodes (Salvador et al., 2005; Achard et al., 2006; Achard and Bullmore, 2007; Liu et al., 2008). In addition to the AAL atlas, other types of anatomical atlases have also been developed for functional connectivity analyses (Lancaster et al., 2000; Maldjian et al., 2003; Faria et al., 2012). The variation in a brain network's topological properties (such as small-worldness), as induced by the variation in nodal definitions, has been a topic of contemporary research interest. In the work of Wang and colleagues (Wang et al., 2009), they conducted a statistical comparison of the topological properties of the functional networks derived from the AAL atlas and another atlas that consists of 70 anatomical regions. While observing robust small-worldness and truncated power-law connectivity degree distributions in both networks, the study found significant differences in multiple topological parameters between the two types of networks. Later, in the work of Zalesky et al. on whole-brain anatomical networks (Zalesky et al., 2010), a significant dependence of brain network properties on the nodal scale was found. Therefore, how the brain is segmented has a great impact on the topological properties of a brain network.

Recently, Wu et al. (2016b) have created a set of atlases that have two types of anatomical definitions (Type I and Type II), each of which consists of five levels of whole brain segmentations with each level linked through ontology-based hierarchical structural relationships (Djamanakova et al., 2014). At the highest ontology level (Level 5), Type I defines seven classical regions of brain ontology [telencephalon (right and left), diencephalon (right and left), mesencephalon, metencephalon, and myelencephalon] and Cerebrospinal fluid (CSF), whereas Type II defines four structures that are more widely used in clinical descriptions (hemispheres (right and left), cerebellum, and brainstem) and CSF (Wu et al., 2016b). Those two types of hierarchical relationships are useful for addressing different clinical hypotheses. For different research purposes, selecting the appropriate type and also the appropriate level of segmentation is of critical importance, especially in fMRI studies. Such multi-granularity atlases, when applied to rs-fMRI based brain network analyses, will thus define 10 types of brain network nodes. Whether the topological properties of a resting state brain network will be affected by those 10 types of multi-granularity nodal definition and how will they be affected are important questions to explore. For example, some network properties may be independent of the ontology relationship type and also the segmentation level, thus suggesting that they are intrinsic brain characteristics in some sense, and other topological properties may be “inherited” from previous levels within the same segmentation type. Examining such factors will greatly further our understanding of rs-fMRI based brain functional networks.

In rs-fMRI based brain network analyses, the typical procedure is to segment a whole brain into multiple distinct anatomical regions based on the structural MRI (e.g., the T1-weighted image) and then co-register the rs-fMRI time series and the structural MRI to define the nodes in all rs-fMRI time series images. The utility of an atlas is its ability to be warped to a subject and thus transfer the structure labels that have been predefined in the atlas to the subject space. In other words, the key is to segment the structural MRIs according to the pre-selected atlas's label definitions. This approach of warping an atlas to a subject is usually referred to as the “single-atlas based segmentation approach.” In recent years, multi-atlas based segmentation methods have gained substantial popularity due to their superior segmentation accuracy (Aljabar et al., 2009; Lötjönen et al., 2011; Wang and Yushkevich, 2013; Wu et al., 2014). In a multi-atlas segmentation approach, instead of using a single atlas, multiple atlases with consistent structural segmentation labels are prepared, warped to the subject image, and then the transformed labels are fused to achieve the best estimation of the structural segmentation for a given subject. In this paper, we segment the structural MRIs in the latter fashion and adopt a fully-automated multi-atlas segmentation pipeline, the diffeomorphic multi-atlas likelihood fusion (MALF) algorithm (Tang et al., 2013), the accuracy of which has been validated on a variety of MRI datasets (Tang et al., 2015).

In this work, we systematically analyze the effects of the multi-granularity, whole-brain segmentation based nodal definitions on the topological architecture of the human brain functional network using the rs-fMRI data from two groups of healthy young subjects. Various topological properties are computed for each network and then compared across levels within the same segmentation type and also between the two different types. Furthermore, we assess the variations across different levels in terms of the degree and betweenness of each node.

The results that we present in this paper will be three-fold: (1) quantitative comparisons of seven topological properties, namely, the average global efficiency, the average local efficiency, the average nodal degree, the average nodal betweenness, the average clustering coefficient, the average characteristic path length, and the small-worldness, across the first three levels of Type I and Type II; (2) quantitative evaluations of two node-specific properties, the nodal degree and the nodal betweenness, across the first three levels of Type I and Type II; (3) comparisons between the corresponding levels of Type I and Type II (e.g., Type I–Level 1 vs. Type II–Level 1) in terms of the small-worldness behavior. We also analyzed the test-retest reproducibility of this multi-granularity network analysis pipeline on a group of 49 healthy young subjects that had been scanned twice.

## Materials and Methods

### Subjects

For the first dataset, a total of 19 subjects (2 males and 17 females, aged 19–25 years with the mean age being 20.95 years) participated in this study at the Brain Imaging Center of South China Normal University. All subjects reported no history of language disabilities, hearing loss, or neurological disorders. Informed consent was obtained from all participating subjects and monetary compensation was allotted for their participation in the experiment. This experiment does not violate any policy or protocol stated by the Institutional Review Board of The First Affiliated Hospital at Sun Yat-sen University of China in accordance with the Code of Ethics of the World Medical Association.

For the test-retest reproducibility experiment, a total of 49 subjects (25 males and 24 females, aged 19–30 years with the mean age being 24.5 years) were involved at Beijing Normal University. This is the same dataset as that used in another reproducibility neuroimaging study, where more details can be found (Lin et al., 2015). All participants completed two fMRI sessions at an average interval of approximately 6 weeks (40.94 ± 4.51 days). All participants were right-handed and had no history of neurological and psychiatric disorders. This dataset has been released in the Consortium for Reliability and Reproducibility (CoRR) database (http://fcon_1000.projects.nitrc.org/indi/CoRR/html/bnu_1.html).

### Data Acquisitions

For the first dataset, all of the imaging data were collected on a Siemens Magnentom 3T Trio Tim MRI scanner (Erlangen, Germany). A three-dimensional (3D) magnetization-prepared rapid gradient-echo (MPRAGE) sequence was used for the structural MRI with a repetition time (TR) of 2.3 s, an echo time (TE) of 3.24 ms, a slice thickness of 1 mm, a voxel size of 1×1×1 mm^3^, a flip angle of 9°, and a field of view (FOV) of 256×256 mm^2^. A 2D echo-planar imaging (EPI) pulse sequence was used for functional images with a TR of 2 s, a TE of 30 ms, a slice thickness of 3.5 mm, a voxel size of 1×1×1 mm^3^, a flip angle of 90°, and a FOV of 224×224 mm^2^. It took 8 min to finish the scanning process with each subject scanned for 240 brain volumes to form the rs-fMRI time series.

For the test-retest experiment dataset, all data were collected using a Siemens Magnentom 3T Trio Tim MRI scanner (Erlangen, Germany) with a 12-channel phased-array head coil at the Imaging Center for Brain Research, Beijing Normal University. A sagittal 3D MPRAGE sequence was used for the 3D structural MRI with a TR of 2.53 s, a TE of 3.39 ms, an inversion time of 1.1 s, a slice thickness of 1.33 mm, a flip angle of 7°, a FOV of 256×256 mm^2^, and 144 sagittal slices covering the whole brain. A 2D EPI pulse sequence was used for functional images with a TR of 2 s, a TE of 30 ms, a slice thickness of 3.5 mm, a matrix size of 64×64, an in-plane resolution of 3.1×3.1 mm^2^, a flip angle of 90°, and a FOV of 200×200 mm^2^. It took about 7 min to finish the scanning process with each subject scanned for 200 brain volumes to form the rs-fMRI time series.

### fMRI Data Preprocessing

All fMRI data were preprocessed using the Statistical Parametric Mapping software (SPM8, http://www.fil.ion.ucl.ac.uk/spm/software/spm8/). The first 10 volumes of each rs-fMRI time series were removed to ensure the subject had entered a stable lying position. After that, the remaining volumes went through slice timing correction followed by head motion correction. These two preprocessing steps are designed to reduce temporal and spatial noise, respectively, in the fMRI data. Finally, every volume of each subject's rs-fMRI data was co-registered to the corresponding T1-weighted structural image. In the co-registration, rigid transformation was employed.

### Multi-Granularity Whole-Brain Segmentation

Segmentation is the final preprocessing step that is essential to region of interest (ROI) based whole brain network analysis. In this study, we employed a validated multi-atlas segmentation algorithm, MALF (Tang et al., 2013), for whole brain segmentations, based on the T1-weighted structural images. Multi-granularity whole-brain segmentations were obtained as guided by two types of hierarchical ontology relationships (Type I and Type II) (Wu et al., 2016b).

**Figure 1:**
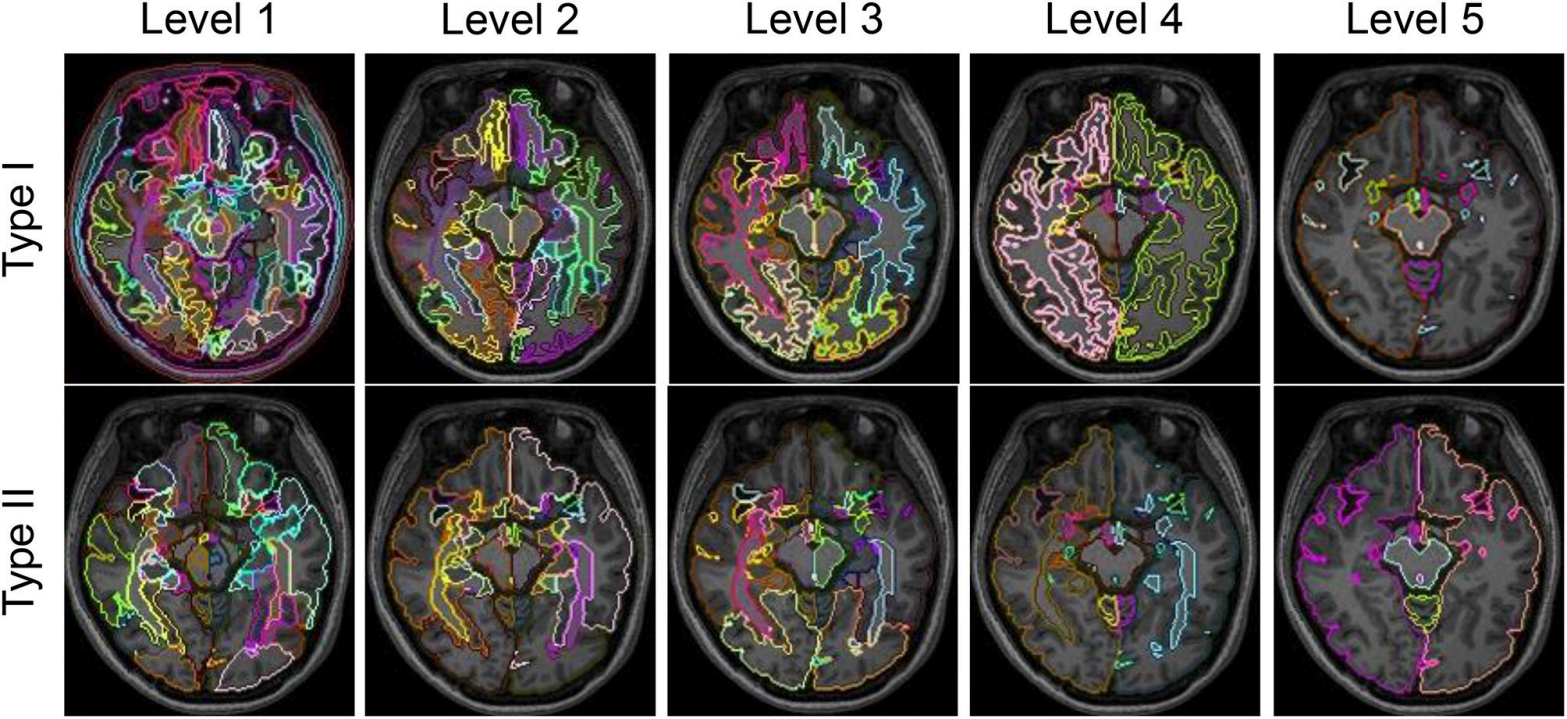
Demonstration of the five granularities of whole-brain segmentations for Type I and Type II in one representative subject at one axial slice.

For each subject, we obtained 10 whole-brain segmentation results that ranged from the finest level, with 281 structures defined, to the coarsest level, having only 5 structures. Type I contains 5 segmentation granularities: 281 structures (Type I–Level 1), 137 structures (Type I–Level 2), 54 structures (Type I–Level 3), 19 structures (Type I–Level 4), and 8 structures (Type I–Level 5). Similarly, Type II has another 5 segmentation granularities: 198 structures (Type II–Level 1), 70 structures (Type II–Level 2), 52 structures (Type II–Level 3), 18 structures (Type II–Level 4), and 5 structures (Type II–Level 5). Figure 1 demonstrates the 10-level whole-brain segmentation results for one representative subject. Further details on these 10 multi-granularity whole-brain segmentations can be found in the Supplementary Material 1 and a previous work (Wu et al., 2016b). In addition, we demonstrate the hierarchical relationship, as described by a structure-relationship table, in Supplementary Material 2. Please note that in the original multi-granularity segmentation, there were non-brain regions defined in Type I–Level 1. In our subsequent network analyses, we have excluded those non-brain regions, resulting in a total of 276 structures for Type I–Level 1.

### Graph Construction

To analyze a brain network using graph theory, we represent it mathematically with nodes and edges, where the nodes are brain regions defined by the multi-granularity segmentation scheme and edges encode the functional connectivity (FC) between every two brain regions. In our work, FC was calculated as the Pearson correlation coefficient (PCC) between every pair of ROIs in terms of their average fMRI intensities. For each subject, all its rs-fMRI images had been co-registered to the structural image, and thus we were able to obtain the average intensity of each fMRI image within any ROI as defined by the whole brain segmentations. For each subject of the first dataset, since there were 230 fMRI time series, a vector of size 230 was created for each ROI. For the reproducibility dataset, a vector of size 190 was created for each ROI. The FC between any pair of ROIs was then quantified by the PCC computed for the corresponding pair of fMRI signal vectors. With 10 granularity levels of segmentations being applied to the 19 subjects' fMRI data, a total of 10×19 = 190 graphs were constructed in this way. For the reproducibility dataset, a total of 10×49×2 = 980 graphs were obtained. The PCCs were then thresholded to build binary networks with a ‘1’ indicating the existence of a connection and a “0” indicating the absence of a connection. The thresholds were chosen to be 0.3, 0.4, 0.5, 0.6, and 0.7, resulting in a total of 950 binary graphs for the first dataset (19×10×5 = 950) and a total of 4,900 binary graphs for the reproducibility dataset (49×2×10×5 = 4900).

**Figure 2:**
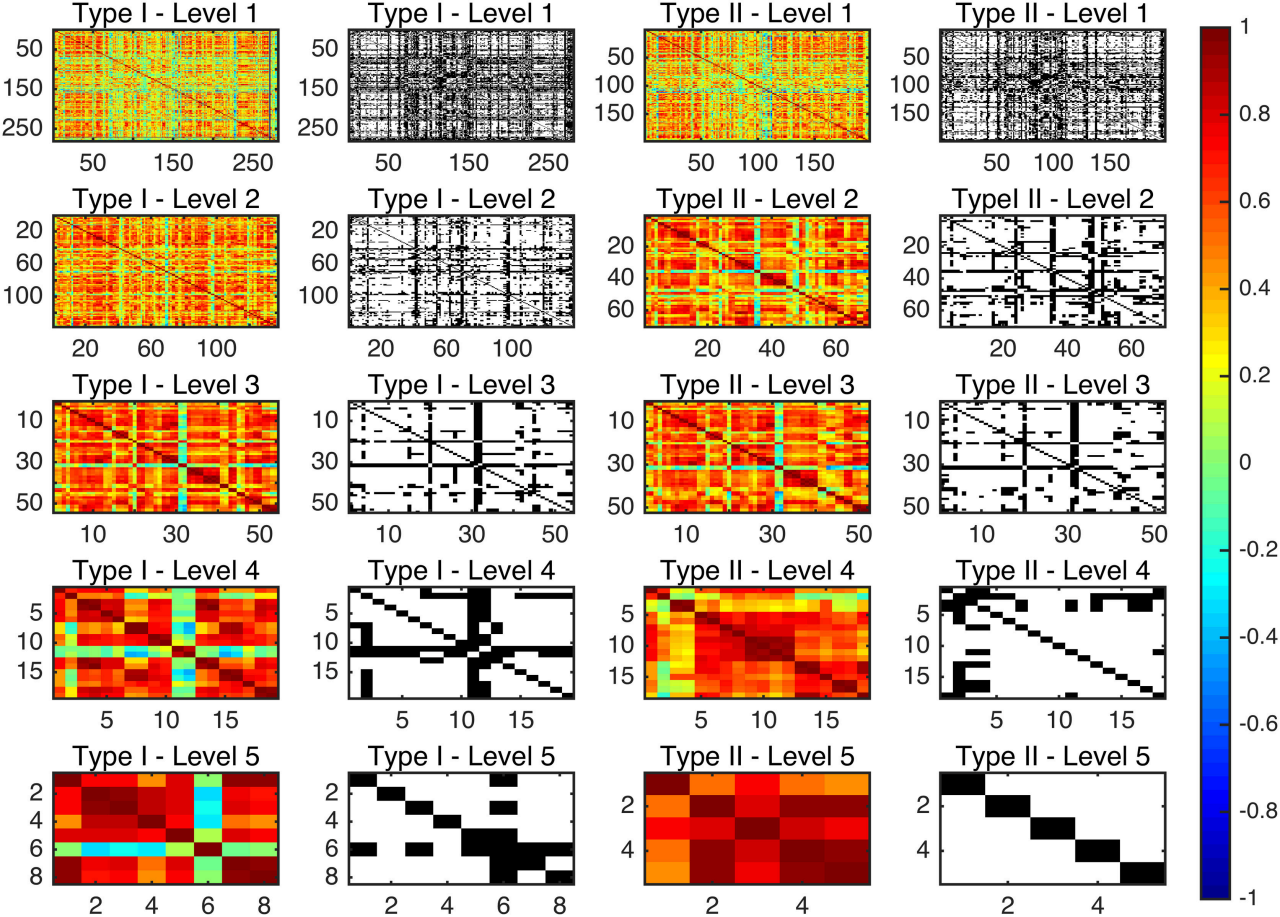
The connectivity matrices before and after binarization, at a PCC threshold of 0.3, for the average functional network for each of the multi-granularity levels in both ontology relationships. The color bar represents the PCC values before binarization (as seen in the 1st and the 3rd columns). White spaces denote the existence of a connection between the corresponding two ROIs after binarization (as seen in the 2nd and the 4th columns).

Since there are very few structures defined in Type I–Level 4 (19), Type I–Level 5 (8), Type II–Level 4 (18), and Type II–Level 5 (5), the graphs at those corresponding levels are relatively sparse after binarization. As such, we did not include graphs based on those four levels in our subsequent graph network analyses but instead focused on the first three levels of both ontology relationships. Figure 2 shows the adjacency matrices before and after thresholding the 10-level whole-brain segmentation based FC matrices of a representative subject, with the threshold for binarization chosen to be 0.3. It is worth noting that our binary graphs do not include self-loops.

### Graph Network Analysis

After creating the binary graphs, we computed topological parameters of interest in three categories: nodal properties, small-world properties, as well as efficiency properties. Here, we will detail the network metrics analyzed in this work with a graph represented as *G*(*N, K*) where *N* denotes the nodes (a total of *n* nodes) and *K* denotes the edges.

#### Nodal Properties

##### Nodal Degree

Nodal degree is the most fundamental network metric and many other topological parameters depend on it. In a graph, the degree of any node is the number of edges that link directly to it, given by $k_{i}=\underset{j\in N}{\sum }a_{ij},i\in N$, where *a*_*ij*_ is the element at the *i*-th row and *j*-th column of the binary matrix representation of the unweighted graph. Nodal degree helps identify hub regions in a network. A node with a high degree is considered to serve much more important roles than nodes with lower degrees. The average nodal degree measures the average number of edges linking to a single node in the network, given by

$K_{nodal}=\frac{1}{n}\underset{i\in N}{\sum }k_{i}=\frac{1}{n\left(n-1\right)}\underset{i,j\in N}{\sum }a_{ij}$.

##### Nodal Betweenness

Similar to nodal degree, nodal betweenness is another nodal property that can also be used for defining hub regions in the network. Nodal betweenness quantifies the importance of a given node in terms of the communication efficiency of the network by calculating the number of the shortest routes between all pairs of nodes that pass through this node, given by *B*_*i*_ = $\underset{m\neq i\neq n\in N}{\sum }\frac{\sigma_{mn}\left(i\right)}{\sigma_{mn}},i\in N$, where σ_*mn*_ is the total number of the shortest paths between nodes *m* and *n*. Among those shortest paths, σ_*mn*_(*i*) denotes the ones that go through node *i*. Nodal betweenness measures the importance of a node's functional capability in sending signals and passing information along all the shortest paths in the brain. The average nodal betweenness measures the centrality of a network and is given by

$B_{nodal}=\frac{1}{n}\underset{i\in N}{\sum }B_{i}$.

#### Small-World Properties

##### Clustering Coefficient

Each node in a network will be assigned a clustering coefficient to quantify its local cliquishness. The clustering coefficient is defined as the number of connections between the direct neighbors of a node in proportion to the maximum possible connections between those neighbors, given by $C_{i}=\frac{2t_{i}}{k_{i}\left(k_{i}-1\right)}$, where *t*_*i*_ denotes the number of triangles whose vertices include node *i* ($t_{i}=\frac{1}{2}\underset{j\in N}{\sum }\underset{h\in N}{\sum }a_{ij}a_{ih}a_{jh}$) and *k*_*i*_ denotes the degree for node *i*. It measures how tightly connected the immediate neighborhood of a node is. The mean clustering coefficient of a network measures the extent of local clustering or cliquishness of the entire network, given by $C_{p}=\frac{1}{n}\underset{i\in N}{\sum }C_{i}$.

##### Characteristic path length

The characteristic path length (or more precisely, the average characteristic path length) is defined as the average length of the shortest path between any pair of nodes, given by $L_{p}=\frac{1}{n\left(n-1\right)}\underset{i\in N}{\sum }\underset{j\neq i\in N}{\sum }d_{ij}$, where *d*_*ij*_ denotes the shortest path length between nodes *i* and *j*. *L*_*p*_ is a global measure of the overall communication efficiency of a network of interest. A shorter path suggests a higher communication efficiency since information flows through fewer edges and thus reaches the destination faster.

##### Small-worldness

Small-worldness is an important notion for characterizing the organizational principles that govern a remarkable variety of social, economical, and biological networks (Latora and Marchiori, 2001). For a network to be considered as possessing the small-worldness property, it should satisfy two conditions: $\gamma =\frac{C_{p}}{C_{rand}}>1$ and $\lambda =\frac{L_{p}}{L_{rand}}\approx 1$, where *C*_*p*_ and *L*_*p*_ are the mean clustering coefficient and mean characteristic path length averaged across all nodes in the objective network and *C*_*rand*_ and *L*_*rand*_ are the mean clustering coefficient and mean characteristic path length of a random network that has the same number of nodes, edges, and nodal degree distribution as the original network but with unrelated topological characteristics. Normally, a random network has a small clustering coefficient and a short characteristic path because of their stochasticity. Hence a small-world network can be quantified as having the property that $S=\frac{\gamma }{\lambda }>1$. Small-worldness is an appealing property that describes the conflicting aspects of a network, the functional segregation and the functional integration of the network.

#### Efficiency Properties

Efficiency is a metric for describing a brain network from a biologically and functionally relevant perspective regarding the information flow within the brain. The efficiency of a network can be evaluated both globally and locally.

##### Global efficiency

The global efficiency measures a network's ability to deliver information at a global scale. It is given by $E_{glob}=\frac{1}{n\left(n-1\right)}\underset{i\in N}{\sum }\underset{j\neq i\in N}{\sum }\frac{1}{d_{ij}}$, where *d*_*ij*_ denotes the shortest path length between nodes *i* and *j*.

##### Local efficiency

The local efficiency quantifies the same property as global efficiency but the quantification is performed within a neighborhood of a node in the network. It is given by $E_{loc}=\frac{1}{n}\underset{i\in N}{\sum }E_{glob}\left(G_{i}\right)$, where *E*_*glob*_(*G*_*i*_) is the global efficiency of *G*_*i*_, the sub-graph composed of the direct neighbors of node *i*.

A summary of the aforementioned seven network metrics is presented in Table 1.

**Table 1:** Description of the seven topological parameters of brain functional networks of interest analyzed in this study.

**Network properties**	**Symbols**	**Descriptions**
Nodal properties	*K*_*nodal*_	The average nodal degree measures the average number of edges linking to a single node
	*B*_*nodal*_	The average nodal betweenness measures the centrality of a network
Small-world properties	*C*_*p*_	The clustering coefficient of a network measures the extent of cliquishness within the network
	*L*_*p*_	The characteristic path length measures the level of overall routing efficiency in the network
	S	Small-worldness, a combination of the clustering coefficient and the characteristic path length, describes the interplay of segregation and integration in the network
Efficiency	*E*_*glob*_	The global efficiency of a network measures the extent of information propagation through the whole network
	*E*_*loc*_	The local efficiency of a network measures the efficiency of information propagation within subnetworks

### Statistical Comparisons

In our graph-based network analysis, we first examined the seven metrics on the average network generated by averaging the fMRI intensities across all subjects of each of the two datasets at each multi-granularity level, with the binary networks themselves obtained by thresholding the PCCs at multiple values-−0.3, 0.4, 0.5, 0.6, and 0.7. We then investigated the individual networks (19 for the first dataset and 49×2 = 98 for the second dataset) at a fixed PCC threshold of 0.3. In each case, we compared first the network properties among the first three levels within the same ontology category (e.g., Type I–Level 1 vs. Type I–Level 2 vs. Type I–Level 3) and then the corresponding levels across ontology categories (e.g., Type I–Level 1 vs. Type II–Level 1). We employed paired Student's *t*-tests to determine the significance of each group comparison. In the case of more than 2 group comparisons, a Bonferroni correction was conducted to address the multiple comparison issue.

For the test-retest reproducibility examination, we analyzed the network metrics of the two rs-fMRI networks for the same subject. Two measures were used to quantify the test-retest reliability. For the first measure, we computed the test-retest difference as $\frac{\left|NM_{1}-NM_{k2}\right|}{0.5\left(\left|NM_{1}|+|NM_{2}\right|\right)}$, where *NM*_1_ denotes the network metric obtained from the first rs-fMRI series and *NM*_2_ denotes the network metric obtained from the second rs-fMRI series of the same subject. For the second measure, we utilized the intra-class correlation coefficient (ICC) (Shrout and Fleiss, 1979). The ICC is defined as $ICC=\frac{BMS-WMS}{BMS+\left(n-1\right)WMS}$, where *BMS* is the between-subject mean square, *WMS* is the within subject mean square and *n* represents the number of repeated observations per subject. ICC describes how strongly units in the same group resemble each other.

## Results

### Average Network Analysis Results

**Figure 3:**
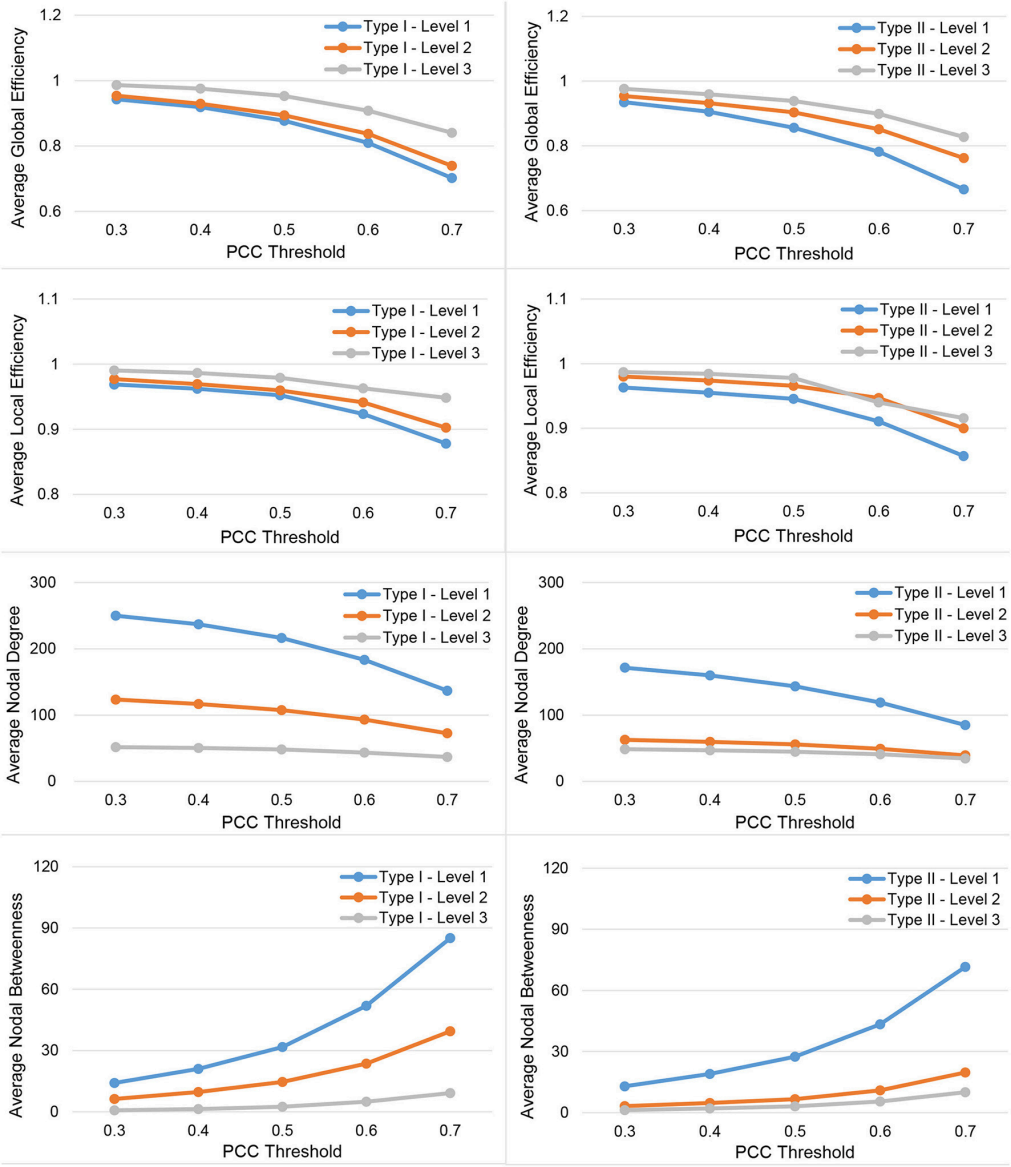
Demonstration of four metrics (the average global efficiency, the average local efficiency, the average nodal degree, and the average nodal betweenness) computed from the average functional network of the 19-subject dataset at various PCC thresholds and granularity levels of Type I and Type II.

**Figure 4:**
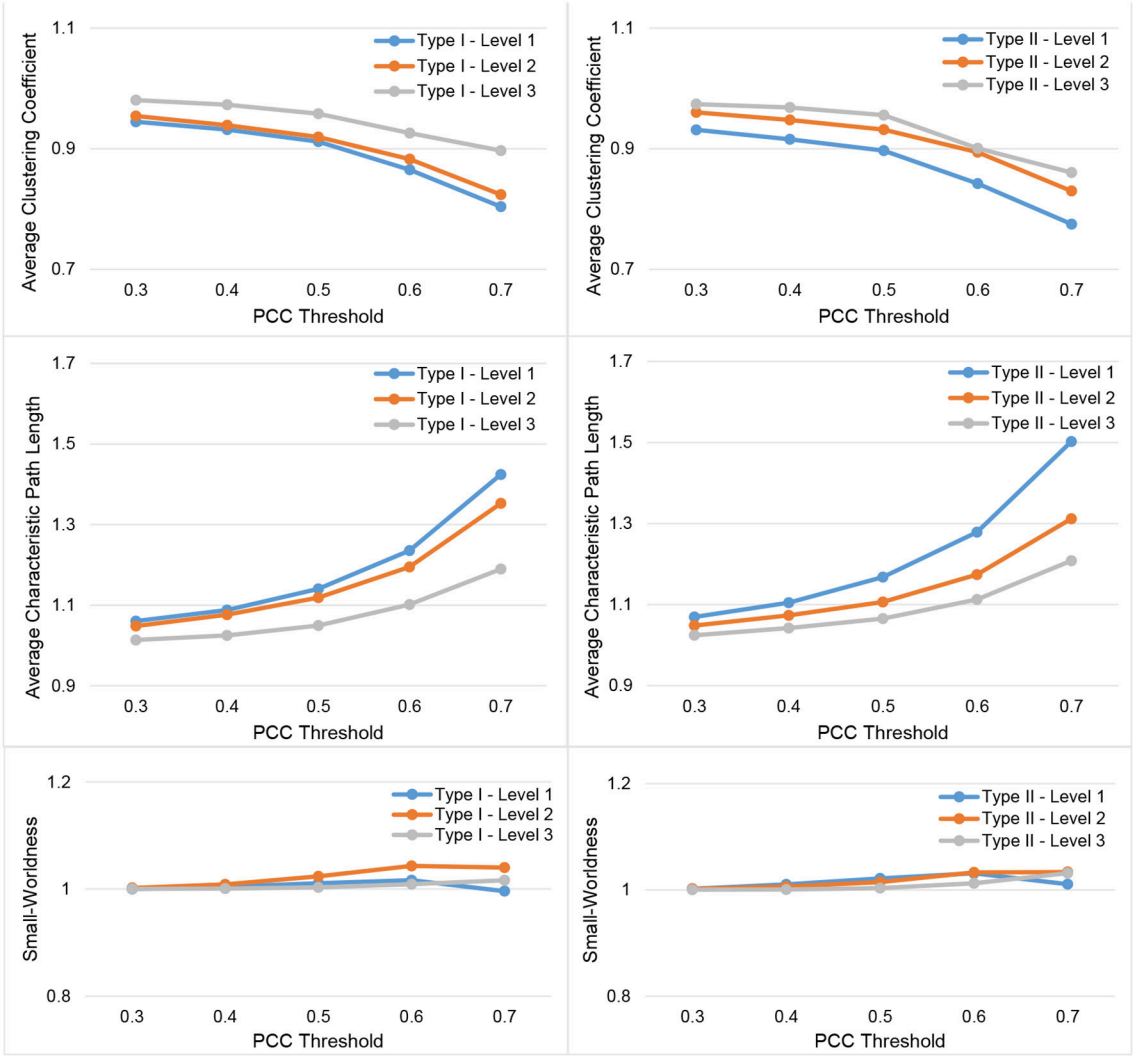
Demonstration of three metrics (the average clustering coefficient, the average characteristic path length, and the small-worldness) computed from the average functional network of the 19-subject dataset at various PCC thresholds and granularity levels of Type I and Type II.

In Figure 3, we show the results for the 19 subjects in terms of four network metrics (the average global efficiency, the average local efficiency, the average nodal degree, and the average nodal betweenness) of the average functional network with nodes defined by the six types of whole-brain segmentations (Type I–Level 1, Type I–Level 2, Type I–Level 3, Type II–Level 1, Type II–Level 2, and Type II–Level 3) at five PCC thresholds (0.3, 0.4, 0.5, 0.6, and 0.7) while the corresponding results for the remaining three metrics (the average clustering coefficient, the average characteristic path length, and the small-worldness) are demonstrated in Figure 4. The corresponding results obtained from the 49 subjects, for both tests, are presented in Figure A1. Consistent patterns are observed; within the same ontology type (Type I or Type II), as the granularity decreases (level 1 to level 2 to level 3), the values of both efficiency metrics (the average global efficiency and the average local efficiency) and the average clustering coefficient increase whereas those of the two nodal property metrics (the average nodal degree and the average nodal betweenness) and the average characteristic path length decrease. For the same type of whole-brain segmentation, as the PCC threshold increases, the values of both efficiency metrics as well as the average nodal degree and the average clustering coefficient decrease whereas the values of the average nodal betweenness and the average characteristic path length increase.

These observations suggest that either a decrease in the granularity with a fixed PCC threshold or an increase in the PCC threshold at a specific granularity level will make the network sparser (less connected, and thus of a smaller average nodal degree). As the segmentation granularity decreases, the binary network becomes less central but more efficient in terms of global, as well as local, information propagation. A coarser granularity level will also induce a network that is more clustered and of a higher overall routing efficiency. In contrast, with a fixed granularity, as the PCC threshold increases, the resultant binary network becomes more central but less efficient globally and locally as well as having less local clusters and being less efficient in terms of overall routing.

The small-world behavior is considered to be significant if the ratio of network localization to network globalization is above 1. In Figure 4 and Figure A2 we observe a small-worldness in all binary networks, obtained, respectively, from the 19 subjects and both tests of the 49 subjects, regardless of the granularity level and the PCC threshold, suggesting the intrinsic nature of the small-worldness of the resting-state brain functional network, especially at a more reasonable PCC threshold such as 0.3 or 0.4.

**Figure 5:**
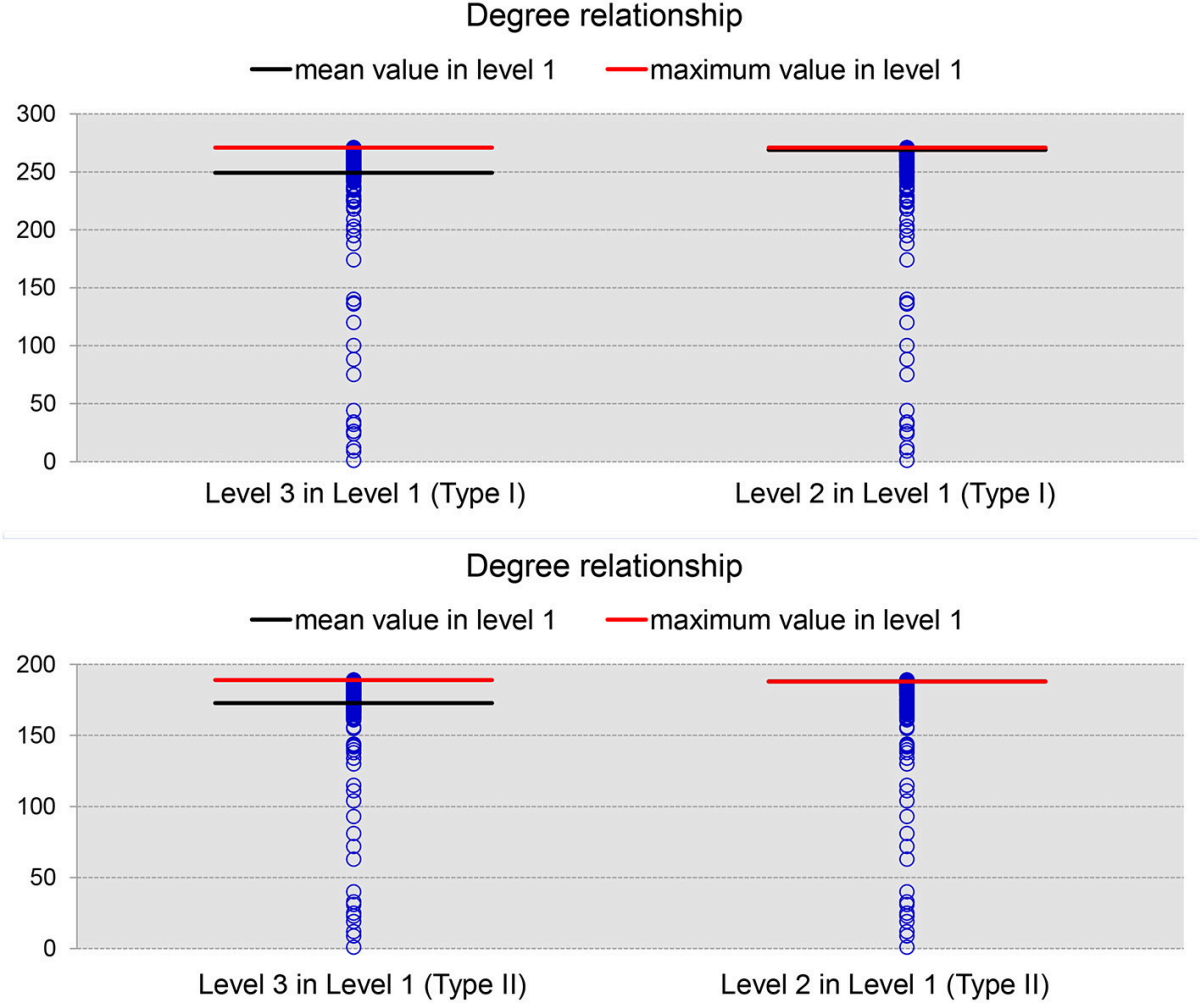
The degree relationships between level 1 and level 3 (left panel) as well as those between level 1 and level 2 (right panel) in Type I (top panel) and Type II (bottom panel). In each panel, the blue circles denote the nodal degrees of all nodes defined in level 1, the black line represents the mean nodal degree values computed across all ROIs belonging to the same, coarser ROI that is in the top 5% of nodal degrees at level 2 (or level 3) while the red line represents the maximum nodal degree value among those top ROIs.

We now turn to our results for the nodal degree and nodal betweenness of the average network binarized at a specific PCC threshold of 0.3. As demonstrated in Figure 5 (results for the 19 subjects), brain regions with large nodal degrees at a finer level will continue to have relatively large nodal degrees when merged into a bigger ROI at a coarser level. In each panel of that figure, the blue circles represent the nodal degrees of all brain regions at Level 1 (Type I or Type II). After identifying the ROIs (nodes) within the top 5% largest nodal degrees at Level 2 and Level 3, we decomposed them into the corresponding ROIs at Level 1 based on the ontology hierarchical relationships. The red line indicates the maximum value and the black line labels the mean value of the nodal degrees of the decomposed ROIs at Level 1. Clearly, the brain regions with top nodal degrees, to some extent, are independent of the granularity-related segmentations; brain regions with large nodal degrees will generally be the same across multiple granularity levels.

**Figure 6:**
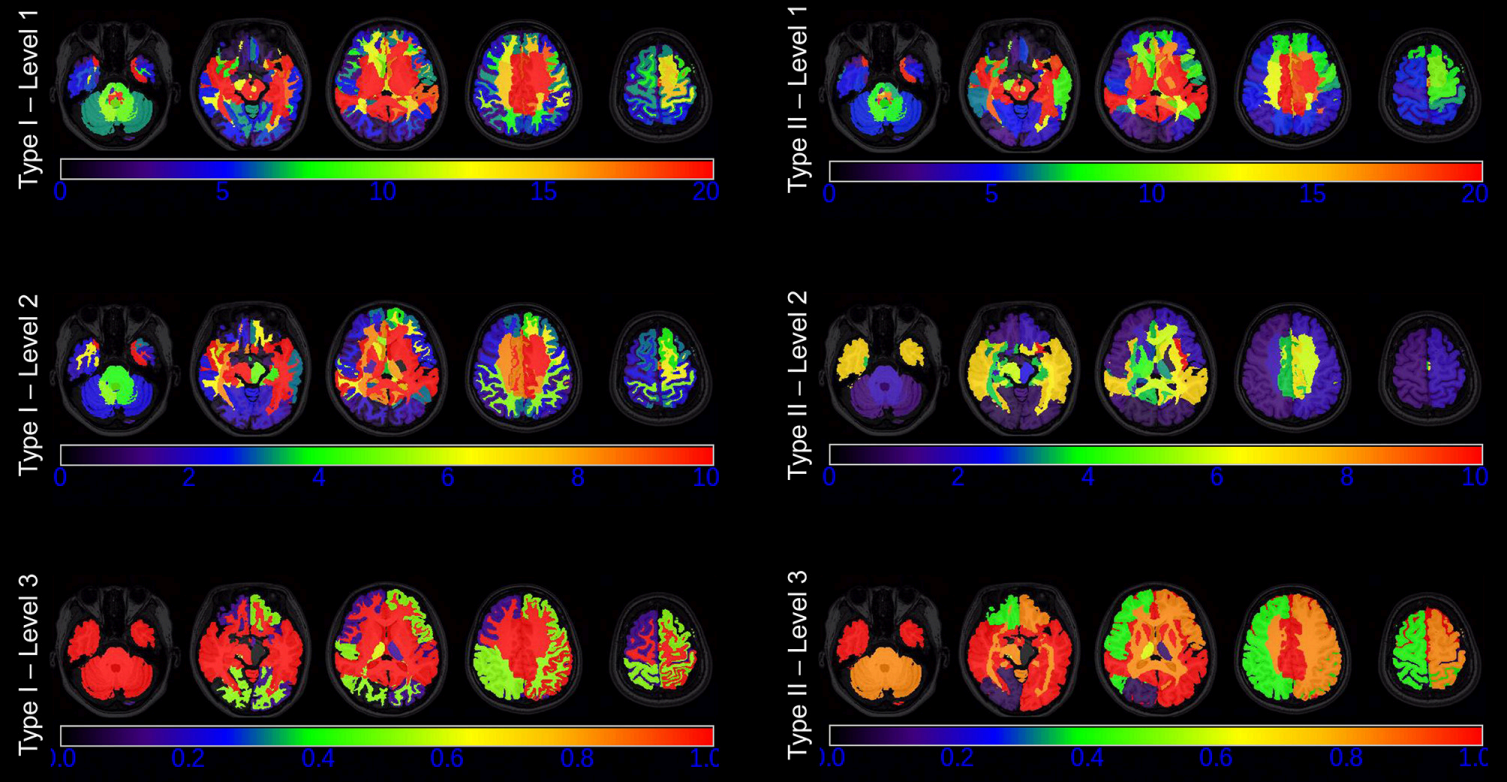
The nodal betweenness of each ROI defined at each of the first three granularity levels in Type I (left panel) and Type II (right panel), as displayed at five axial slices. Note that the range of the color bar varies from level to level.

Figure 6 shows the color maps of nodal betweenness for each ROI defined at each granularity level in both Type I and Type II at the PCC threshold of 0.3. We found that ROIs centrally located within the brain have much higher betweenness than those on the periphery of all three granularity levels in the two types of segmentations, indicating that regions in the center of the brain carry more responsibility for whole brain communication and that such a property is not specific to any segmentation scheme. For different ROIs, the nodal betweenness distribution patterns are similar across different granularity levels as well as different ontology types in the sense that brain regions with a large or a small nodal betweenness generally coincide across the six types of networks as defined by the six types of whole-brain segmentations.

### Individual Network Analysis Results

**Figure 7:**
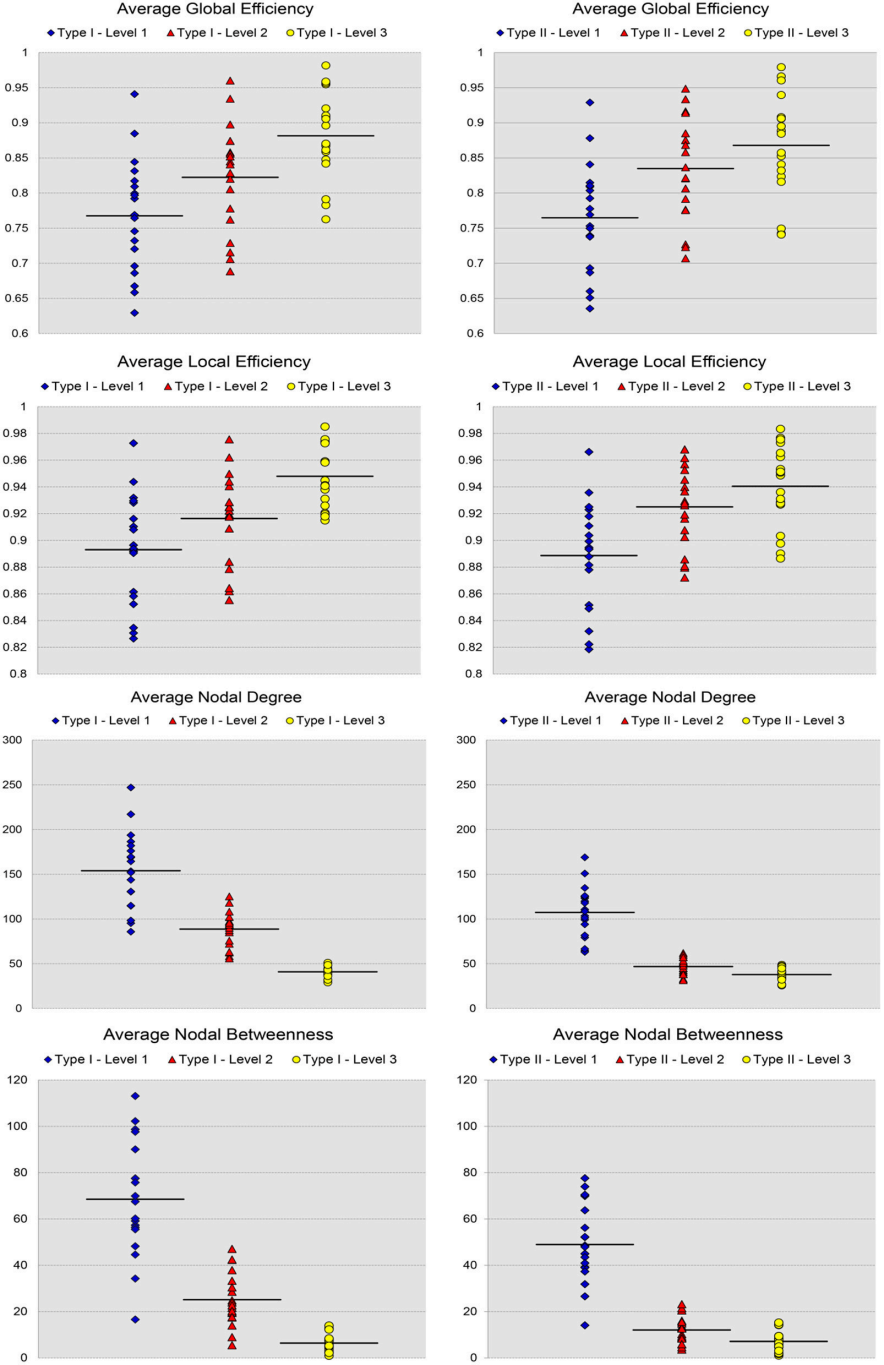
Scatter plots of the 19 values for certain network metrics (the average global efficiency, the average local efficiency, the average nodal degree, and the average nodal betweenness), as well as their mean values, on the first three levels of both types of the 19-subject dataset.

**Figure 8:**
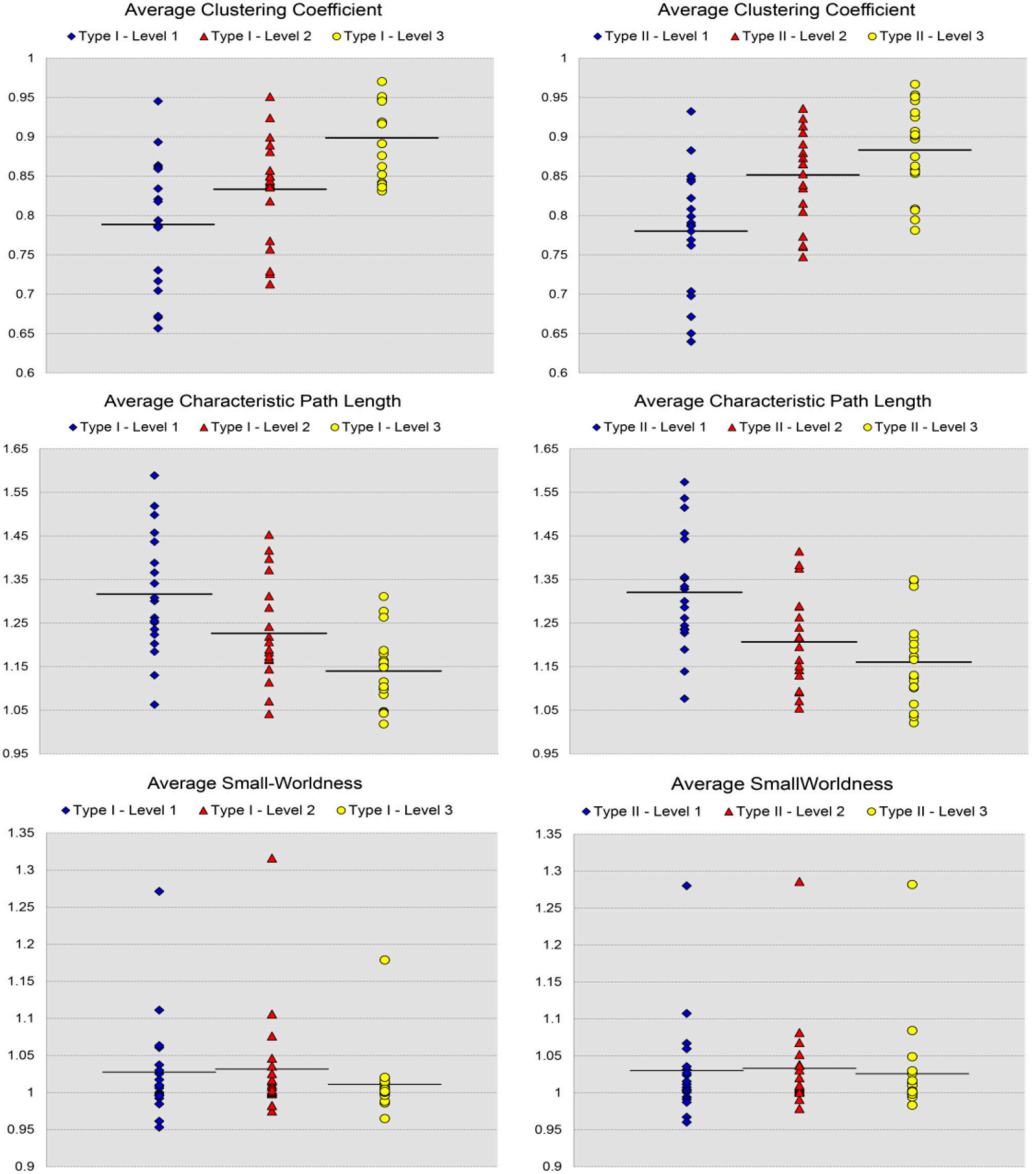
Scatter plots of the 19 values for individual network metrics, in terms of the average clustering coefficient, the average characteristic path length, and the small-worldness, as well as their mean values, computed from the first three levels of both Type I and Type II of the 19-subject dataset.

For the analysis of the subject-based individual networks, we investigated at a fixed PCC threshold of 0.3. In Figure 7, we show the values of four network metrics (the average global efficiency, the average local efficiency, the average nodal degree, and the average nodal betweenness) on each of the 19 functional networks for the first dataset as well as the means computed across those 19 values, with nodes defined by the six types of whole-brain segmentations (Type I–Level 1, Type I–Level 2, Type I–Level 3, Type II–Level 1, Type II–Level 2, and Type II–Level 3). The corresponding results for the 98 networks from the reproducibility dataset are demonstrated in Figure A3. Meanwhile, the corresponding results on the other three metrics (the average clustering coefficient, the average characteristic path, and the small-worldness) are demonstrated in Figure 8 and Figure A4. The mean and standard deviations of those seven metrics, computed across all individual networks of the 19 subjects and both tests of the 49 subjects, are tabulated, respectively in Table 2 and Table A1. Within the same ontology type, the group differences between any two levels (for example, Level 1 vs. Level 2), in terms of all network metrics except the small-worldness, are statistically significant with a *p*-value less than 1*e*^−6^ in all cases for both datasets. This again supports our previous conclusion that as the segmentation granularity decreases, the binary network becomes less connected, less central, more efficient in information propagation both globally and locally, more clustered, as well as of a higher overall routing efficiency, all of which are statistically significant even after multiple comparison correction. For the small-worldness, all individual networks have a small-worldness larger than or approximately equal to 1, indicating a presence of small-worldness in all networks. We observed *p*-values larger than 0.04 for the comparisons of every two levels of Type I and *p*-values larger than 0.01 for the comparisons of every two levels of Type II for the 19-subjects dataset. For the reproducibility dataset, the *p*-values were, respectively larger than 0.1 and 0.01 for the comparisons of every two levels of Type I and Type II for both tests. Such results indicate no significant group differences after Bonferroni correction across different levels within the same ontology relationship. Comparing the small-worldness between Type I and Type II, we obtained a *p*-value of 0.152, 0.999, 0.999 between the two Level 1 classes, 0.774, 0.999, 0.385 between the two Level 2 classes, and 0.051, 0.676, 0.672 between the two Level 3 classes, for the first dataset and the two tests in the reproducibility experiment. Therefore, we have further evidence that small-worldness is unaffected by the multi-granularity segmentation and is an intrinsic property of healthy young human brains.

**Table 2:** The mean and standard deviations of the seven network metrics of interest, for the three granularity levels in Type I and Type II, as computed across the 19 individual networks.

	**Type I-Level 1**	**Type I-Level 2**	**Type I-Level 3**
Average global efficiency	0.768 ± 0.081	0.822 ± 0.076	0.881 ± 0.061
Average local efficiency	0.893 ± 0.041	0.916 ± 0.034	0.948 ± 0.022
Average nodal degree	153.911 ± 42.534	88.659 ± 19.696	40.957 ± 6.119
Average nodal betweenness	68.514 ± 24.747	25.134 ± 11.407	6.396 ± 3.697
Average clustering coefficient	0.789 ± 0.080	0.833 ± 0.067	0.899 ± 0.045
Average characteristic path length	1.316 ± 0.139	1.226 ± 0.117	1.140 ± 0.081
Small-worldness	1.027 ± 0.069	1.032 ± 0.076	1.011 ± 0.043
	**Type II-Level 1**	**Type II-Level 2**	**Type II-Level 3**
Average global efficiency	0.765 ± 0.077	0.835 ± 0.072	0.868 ± 0.072
Average local efficiency	0.889 ± 0.039	0.925 ± 0.031	0.941 ± 0.031
Average nodal degree	107.303 ± 28.678	46.830 ± 9.309	37.897 ± 6.964
Average nodal betweenness	48.937 ± 16.817	12.037 ± 5.659	7.117 ± 4.161
Average clustering coefficient	0.780 ± 0.079	0.852 ± 0.061	0.883 ± 0.057
Average characteristic path length	1.320 ± 0.135	1.207 ± 0.107	1.160 ± 0.101
Small-worldness	1.030 ± 0.070	1.033 ± 0.067	1.026 ± 0.066

### Reproducibility Results

The test-retest reproducibility results at the threshold of 0.3, in terms of both differences and ICC values, for the seven metrics, are tabulated in Tables 3, 4. Clearly, low test-retest differences and large ICC values have been obtained, indicating a strong reproducibility and robustness in our multi-granularity functional network analysis pipeline. Among all seven metrics, the average nodal betweenness had relatively lower reproducibility. The corresponding reproducibility results at other threshold values (namely 0.4, 0.5, 0.6, and 0.7) are listed in Tables A2–A9. At other threshold values, strong reproducibility has also been observed.

**Table 3:** The mean and standard deviations of the test-retest differences, in terms of the seven network metrics of interest, for the three granularity levels in Type I and Type II, as computed across the 49 subjects involved in the reproducibility experiment at a threshold of 0.3.

	**Type I-Level 1**	**Type I-Level 2**	**Type I-Level 3**
Average global efficiency	0.011 ± 0.008	0.012 ± 0.008	0.011 ± 0.012
Average local efficiency	0.024 ± 0.017	0.03 ± 0.021	0.026 ± 0.021
Average nodal degree	0.05 ± 0.033	0.06 ± 0.038	0.04 ± 0.028
Average nodal betweenness	0.071 ± 0.063	0.082 ± 0.4	0.049 ± 0.285
Average clustering coefficient	0.019 ± 0.012	0.012 ± 0.009	0.016 ± 0.013
Average characteristic path length	0.027 ± 0.019	0.004 ± 0.014	0.013 ± 0.042
Small-worldness	0.005 ± 0.004	0.012 ± 0.009	0.028 ± 0.023
	**Type II-Level 1**	**Type II-Level 2**	**Type II-Level 3**
Average global efficiency	0.009 ± 0.007	0.0683 ± 0.04	0.008 ± 0.01
Average local efficiency	0.02 ± 0.018	0.028 ± 0.021	0.023 ± 0.019
Average nodal degree	0.041 ± 0.036	0.046 ± 0.032	0.037 ± 0.026
Average nodal betweenness	0.082 ± 0.39	0.085 ± 0.39	0.011 ± 0.012
Average clustering coefficient	0.028 ± 0.026	0.012 ± 0.011	0.019 ± 0.012
Average characteristic path length	0.023 ± 0.02	0.009 ± 0.031	0.014 ± 0.043
Small-worldness	0.2 ± 0.147	0.022 ± 0.016	0.038 ± 0.023

**Table 4:** The ICC values of the seven network metrics of interest, for the three granularity levels in Type I and Type II, as computed across the 49 subjects involved in the reproducibility experiment at a threshold of 0.3.

	**Type I-Level 1**	**Type I-Level 2**	**Type I-Level 3**
Average global efficiency	0.942	0.958	0.81
Average local efficiency	0.945	0.974	0.702
Average nodal degree	0.956	0.975	0.824
Average nodal betweenness	0.73	0.972	0.998
Average clustering coefficient	0.966	0.98	0.815
Average characteristic path length	0.944	0.968	0.745
Small-worldness	0.962	0.943	0.962
	**Type II-Level 1**	**Type II-Level 2**	**Type II-Level 3**
Average global efficiency	0.82	0.829	0.823
Average local efficiency	0.869	0.856	0.835
Average nodal degree	0.938	0.812	0.931
Average nodal betweenness	0.813	0.802	0.831
Average clustering coefficient	0.872	0.916	0.997
Average characteristic path length	0.865	0.905	0.913
Small-worldness	0.889	0.956	0.996

## Discussion

In this study, we investigated the influence of a recently-developed, multi-granularity, whole-brain segmentation scheme on the topology of resting-state brain functional networks in two healthy young populations. A total of six types of functional networks have been analyzed, with the network nodes defined according to two ontology segmentation types each of which includes three hierarchical levels (Djamanakova et al., 2014; Wu et al., 2016b).

According to our results, the nodal degree and the nodal centrality on average decreased significantly as the segmentation's granularity decreased. However, both the nodal degree and the nodal betweenness distribution patterns, in terms of their values on nodes within the same network, are consistent across different levels. As presented in Figure 5, the brain regions with the largest nodal degree at one level will also rank highly among the other two levels in terms of nodal degree. Similar patterns were also observed for the nodal betweenness (see Figure 6), and the central-to-peripheral patterns were consistent across different levels and across ontology types. These observations are in line with previous findings on the relationship between nodal scale and the two nodal properties in anatomical networks (Zalesky et al., 2010). Our results suggest that as the granularity changes, the absolute values of nodal degree and nodal betweenness change accordingly but the relative values within a single network do not change considerably. Another important conclusion drawn from our observations is that the average nodal degree is more related to the sparsity of the network than the granularity whereas the average nodal betweenness is more affected by the granularity than the general sense of sparsity. As shown in our analyses of the average network, the average nodal degree decreased when the network became sparser, whether it be by a decrease in the granularity or by an increase in the PCC threshold. However, the average nodal betweenness decreased when the network became sparser as induced by a granularity decrease but it increased when the network became sparser due to a PCC threshold increase. This indicates that the nodal betweenness was more affected by the nodal definitions than the binarization process. This conclusion applies to the two efficiency metrics (the average global efficiency and the average local efficiency), the average characteristic path length, and the average clustering coefficient as well.

Within the same ontology relationship type, as the granularity decreased, the network became more efficient at information propagation, as evaluated by the four efficiency metrics—the average global efficiency, the average local efficiency, the average characteristic path length, and the average clustering coefficient. This observation agrees with our hypothesis that the ROIs will be better placed to “communicate” after merging multiple small ROIs into single larger ones. Similar brain network patterns have been revealed in previous studies (Zalesky et al., 2010). A dependence of those four network metrics on the whole-brain segmentation type has also been reported in the work of Wang and colleagues (Wang et al., 2009).

One of the most important findings emerging from this study is that the six types of brain functional networks all exhibited small-worldness and there is no significant group difference among levels within the same type nor the corresponding levels across the two types (e.g., Type I–Level 1 vs. Type II–Level 1). This indicates an intrinsic nature to the small-worldness of brain functional networks in the healthy young population. Evidence exists on the small-worldness of brain functional network using both the AAL atlas (Salvador et al., 2005; Achard et al., 2006) and another atlas consisting of 70 anatomical regions for nodal definition (Wang et al., 2009).

The multi-granularity, hierarchical, whole-brain segmentations have been widely used in a variety of neuroimaging studies (Djamanakova et al., 2014; Liang et al., 2015; Ma et al., 2015; Wu et al., 2016a,b). However, to the best of our knowledge, this is the first time that they have been applied to whole-brain resting-state functional network analyses using graph theory. This is also the first time that the topological architecture of the resting-state brain functional network, which, in this case, was in terms of seven metrics, has been systematically analyzed with regards to the relationship to those hierarchical segmentations. Such a quantitative evaluation of the network properties for brain networks built upon those multi-granularity segmentations provides valuable resources and guidance for brain network analyses with new types of nodal definitions other than the typically used AAL atlas.

Overall, strong test-retest reproducibility of this multi-granularity network analysis pipeline has been observed from our experiments. That said, our ICC results varied depending on the threshold used, the segmentation type and level, as well as the network metric of interest. We conjecture that using the correlation coefficient threshold may have also contributed somehow to the apparent quality of the results.

In this paper, the nodal definitions of each individual network were determined based on a sophisticated multi-atlas segmentation algorithm (Tang et al., 2013) instead of the normally used single-atlas based segmentation methods. This ensures that the anatomical meaning of each node in the brain network is more accurate, which will likely reduce the noise extracted from the fMRI time series and unveil the true brain network patterns.

An interesting question is as follows, “which ontology type should I use for my network analysis?” Such a selection should depend on the research goal to determine which one (Type I or Type II) is more appropriate. At the lowest ontology level, the brain parcellation defined in Type I is more classical in brain ontology, whereas that in Type II is more widely used in clinical descriptions. For example, the metencephalon defined in Type I includes pons and cerebellum sharing the same developmental precursor, whereas the brainstem and cerebellum (each of which is defined as a single unit in Type II) are often defined as different entities in brain imaging research.

Within the same ontology relationship type, we may ask, “which granularity level will best serve a study's purpose?” This is a challenging yet interesting question to explore. At a level of lower granularity, the ROIs will be defined in a coarser fashion which will increase the imprecision when calculating the connectivity strength, given that it is computed as the correlation between the average fMRI intensities within each defined ROI. The larger the ROI, the less detail and less precision the network will possess. However, a finer granularity level will decrease segmentation accuracy (achieving a high segmentation accuracy at the finest level of image voxel is the most challenging) and reproducibility (Djamanakova et al., 2014). The middle level, Level 3, is generally considered to have a good balance between accuracy and precision (Djamanakova et al., 2014) and has been shown to possess good disease detection power (Luo and Tang, 2017). Regarding the utility in brain network analysis, as observed from our experiments, level selection would not significantly affect the final clinical conclusions drawn as long as the segmentation level is consistently applied to all images involved. With that being said, more substantial analyses need to be done to explore the most “appropriate” granularity level for rs-fMRI based brain network analyses.

There are several limitations to this study. (1) The network binarization process was conducted based on thresholding the PCCs at specific threshold values. In the individual network analyses, we empirically selected the threshold to be 0.3. There is an alternative approach that involves controlling the sparsity and wiring cost of the network as threshold measurements (Newman, 2003; He et al., 2008; Wang et al., 2009), which we would expect to be more rigorous than our approach. We will examine the possibility of using this solution in our future work. (2) In our analysis of the two node-specific properties, nodal degree and nodal betweenness, we have only investigated the distribution patterns and compared across granularity levels qualitatively. It would be illuminating to evaluate those properties quantitatively and statistically. (3) In this work, we conducted a standard set of simple rs-fMRI preprocessing steps using SPM8 (Braun et al., 2012; Liu et al., 2013; Woo et al., 2014). Recently, more advanced rs-fMRI denoising techniques, such as band-pass filtering, more advanced approaches for head motion correction, and scrubbing, have been proposed (Dipasquale et al., 2017; Parkes et al., 2018). Appropriate preprocessing is of great importance for fMRI analyses. And thus, it is critical to test whether the conclusions drawn from this study will still hold when employing more advanced preprocessing techniques, a task that we expect will be one of our future endeavors. (4) Lastly but most importantly, the multi-granularity whole-brain segmentations have hierarchical relationships, which in some sense forms a network themselves. How to effectively integrate the network information from those multiple levels to jointly analyze the brain functional network is a scientific question of great importance that may further our understanding of the architecture of the human brain and also provide network-based neuro-informatics of critical utility in disease detection and prediction.

The focus of this study is foremost to provide a guidance for researchers who want to use the multi-granularity segmentation pipeline for rs-fMRI analyses, rather than the typically used AAL atlas. We believe this work will be a timely and appropriate addition to the existing literature regarding how to use the multi-granularity whole-brain segmentations in rs-fMRI studies.

## Author Contributions

YG and HW carried out the fMRI data preprocessing and all statistical analyses. JL conducted the multi-granularity whole brain segmentations. NW contributed to the interpretation of the experimental results. HL contributed to data collection and result interpretation. XT contributed to the conceptualization and design of the study and provided critical feedback on the manuscript. All the authors read and contributed to the manuscript and the ideas presented in it.

### Conflict of Interest Statement

The authors declare that the research was conducted in the absence of any commercial or financial relationships that could be construed as a potential conflict of interest.
