# Supplementary material for: Multi-Granularity Whole-Brain Segmentation Based Functional Network Analysis Using Resting-State fMRI
Source: Front Neurosci. 2018 Dec 12;12:942. doi: 10.3389/fnins.2018.00942 (PMC6299028; doi:10.3389/fnins.2018.00942)
Supplement: Supplementary file 1 [file Data_Sheet_1.DOCX]

**Description of the multi-granularity hierarchical segmentation:** The smallest unit of an image one can define is one voxel. The most natural way to join voxels and define a structure is to use a hypothesis- or biology- driven approach. The goal of the multi-granularity hierarchical segmentation system was to introduce an approach to the systematic management of anatomical structures by using a hierarchical structural relationship based on ontology. For example, certain brain atlases and the general anatomical literature (1,2) have defined how various structures should be hierarchically grouped. Based on the minimum units (image voxels), higher hierarchical levels (superstructures) can be defined by joining the units, according to (3). The following figure shows images of the hierarchical relationship defined in five different levels (courtesy of (4)). For more details, please refer to (4) and the structure-relationship table listed in Supplementary Material 2.


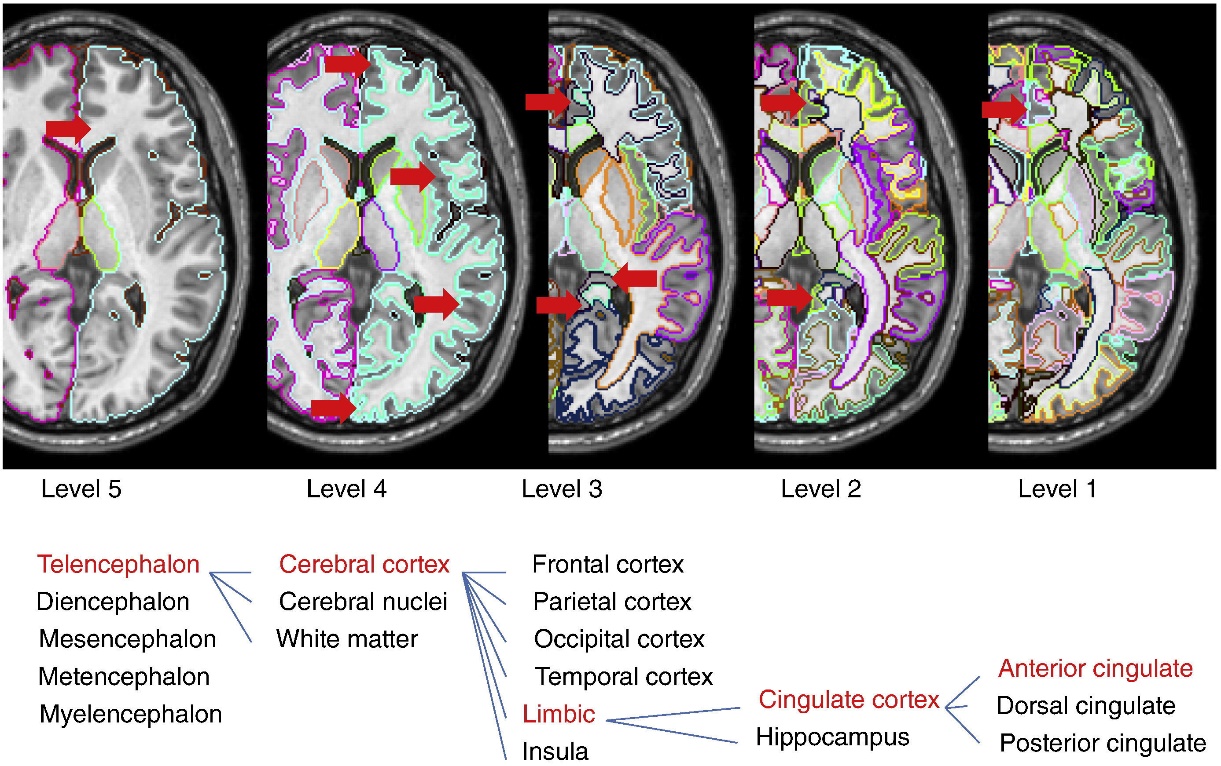


**Figure.** Hierarchical relationship defined at five different levels. Top panel: An image representation of the five-level structural delineation. Bottom panel: An example of the hierarchical definitions in the telencephalon at level 5 to the anterior/dorsal/posterior cingulate cortex at level 1. The structures with red names are indicated in the images, portraying how finer substructures are defined as the level increases. (Figure courtesy of (5)).

(1) Mai JK, Paxinos G, Voss T. Atlas of the Human Brain. Academic Press, San Diego. (2007)

(2) Puelles L, Harrison M, Paxinos G, Watson C. A developmental ontology for the mammalian brain based on the prosomeric model. *Trends in neurosciences* (2013) **36**:10:570-578.

(3) Mai JK, Paxinos G, Voss T. Atlas of the Human Brain. 3rd Edition. Academic

Press.

(4) Wu D, Ma T, Ceritoglu C, Li Y, Chotiyanonta J, Hou Z, et al. Resource atlases for multi-atlas brain segmentations with multiple ontology levels based on T1-weighted MRI. *Neuroimage* (2016) **125**:120-30 doi: S1053-8119(15)00953-2 [pii].


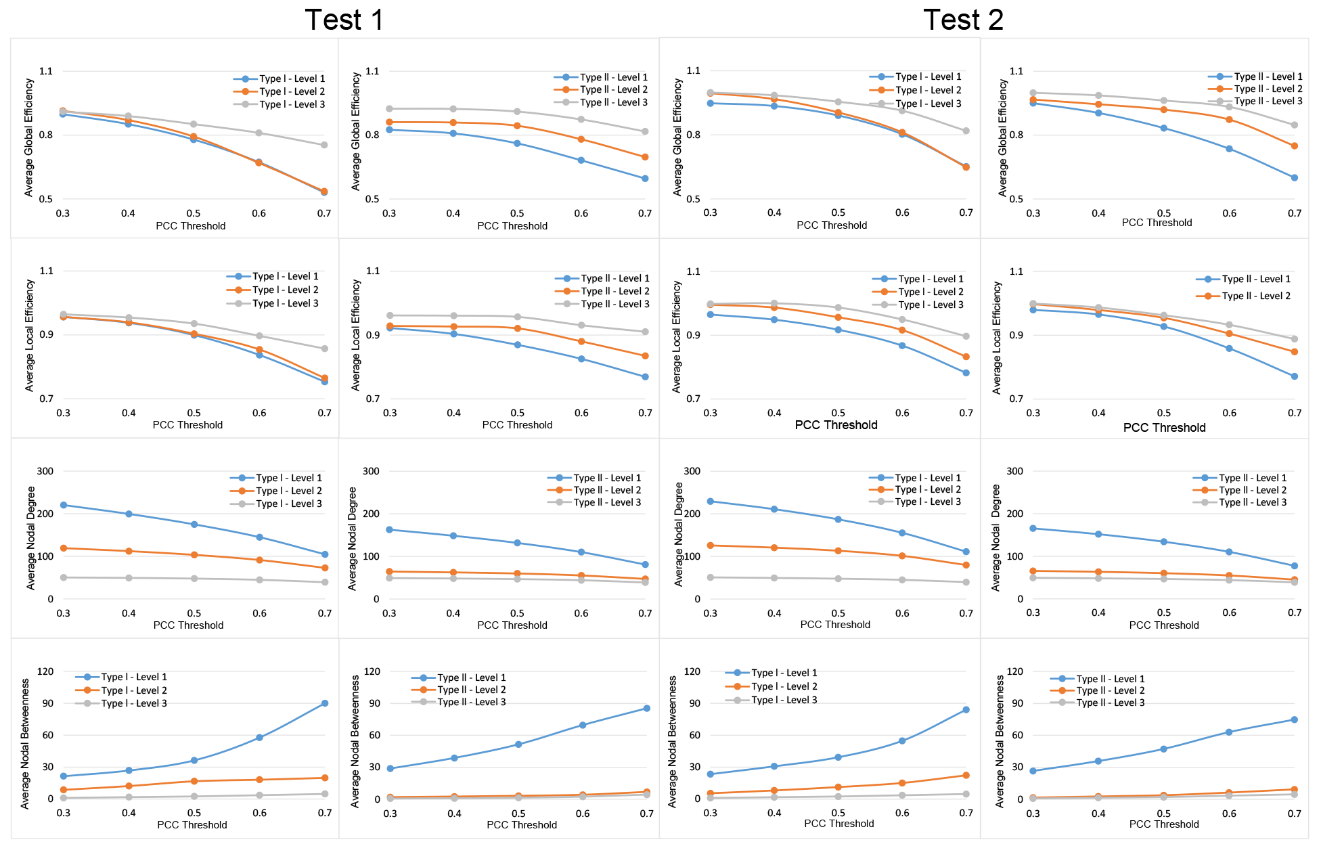


**Figure A1.** Demonstration of four metrics (the average global efficiency, the average local efficiency, the average nodal degree, and the average nodal betweenness) computed from the average functional network of both tests of the reproducibility dataset at various PCC thresholds and granularity levels of Type I and Type II.


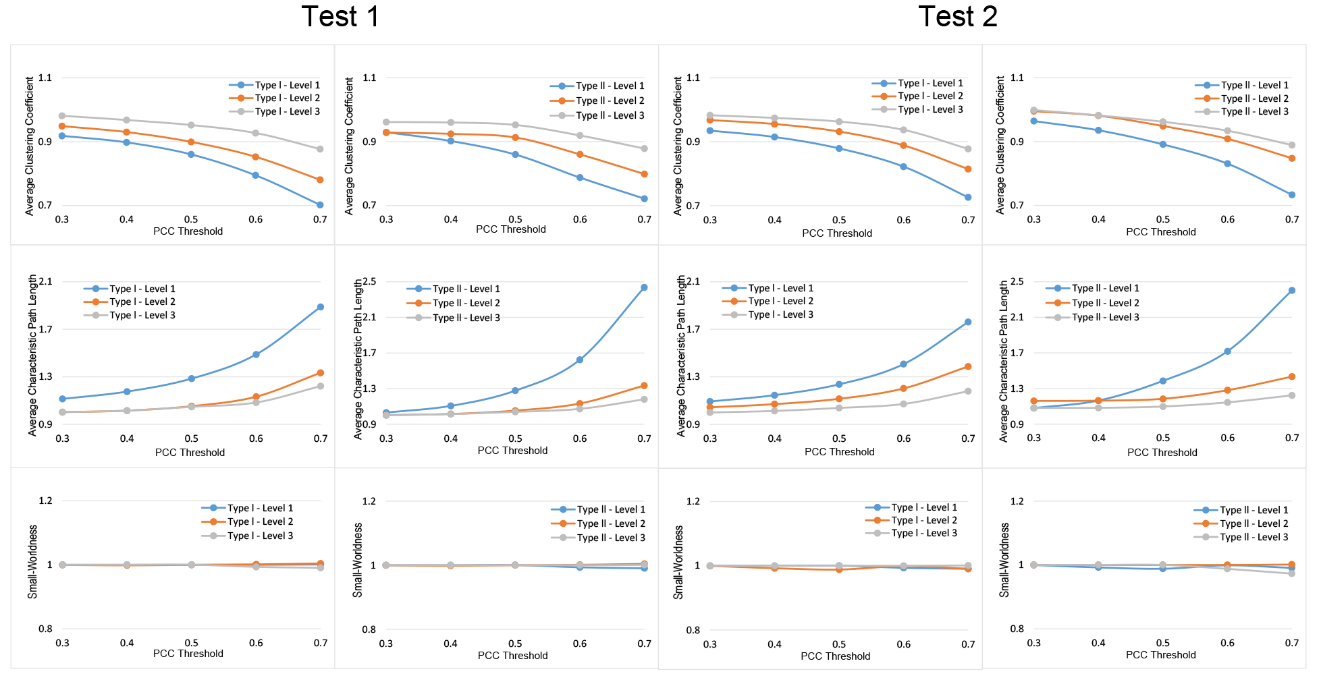


**Figure A2.** Demonstration of three metrics (the average clustering coefficient, the average characteristic path length, and the small-worldness) computed from the average functional network of both tests of the reproducibility dataset at various PCC thresholds and granularity levels of Type I and Type II.


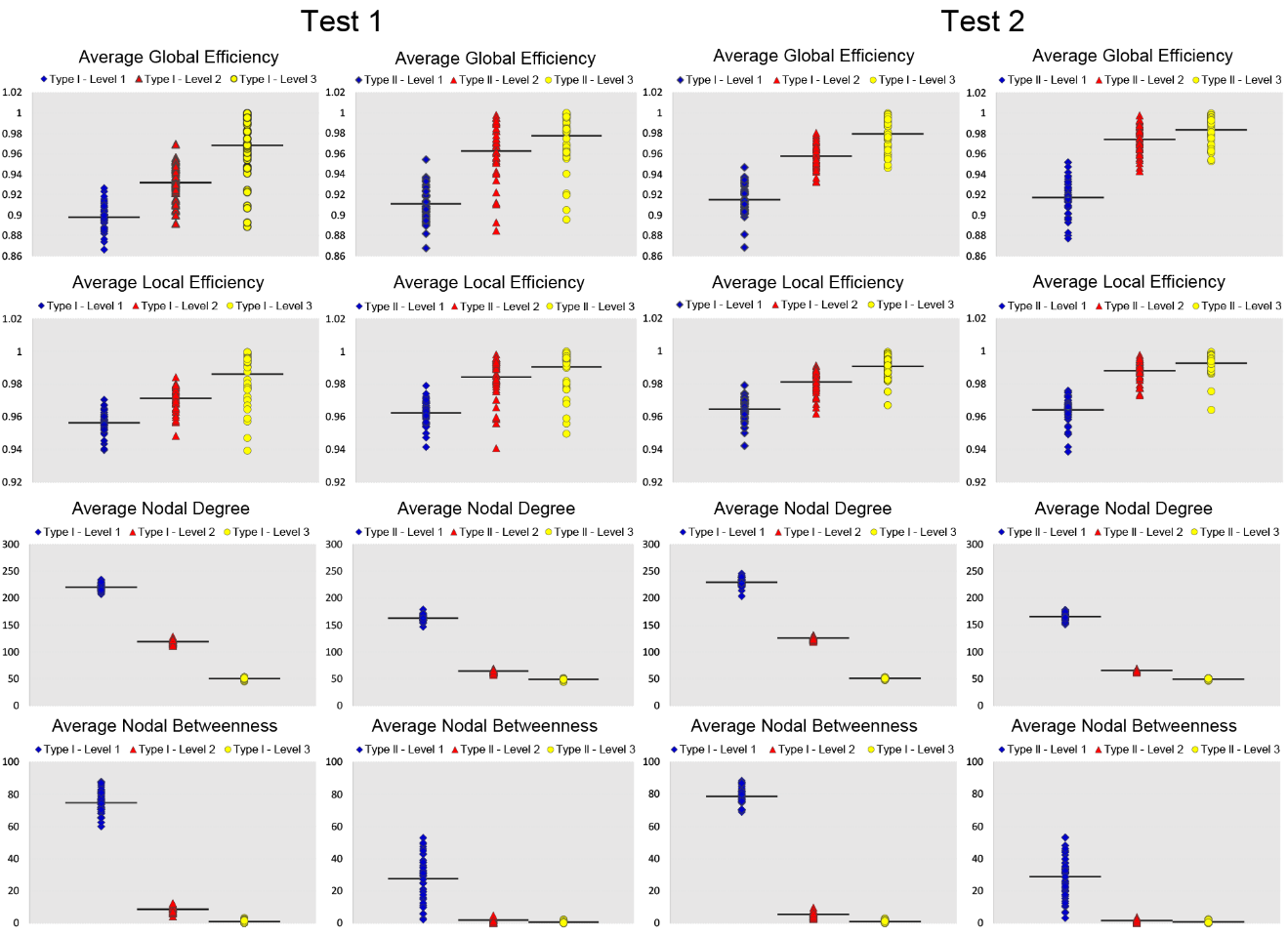


**Figure A3.** Scatter plots of the 49 values for certain network metrics (the average global efficiency, the average local efficiency, the average nodal degree, and the average nodal betweenness), as well as their mean values, of the first three levels of both types of the reproducibility dataset.


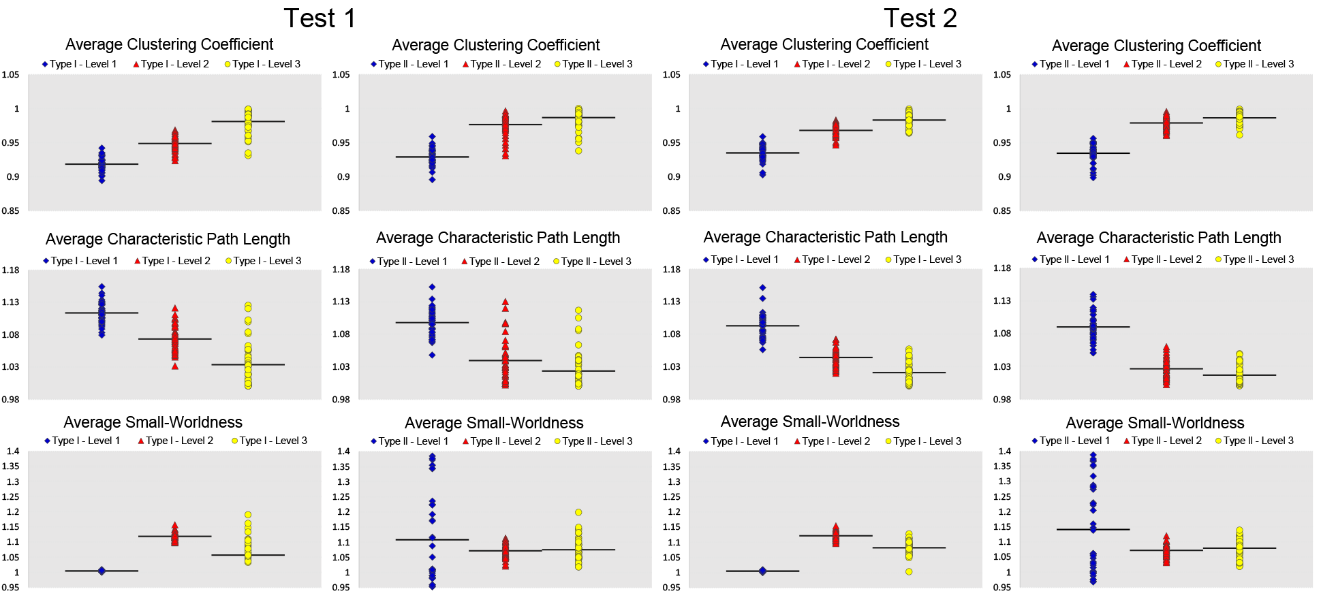


**Figure A4.** Scatter plots of the 49 values for individual network metrics, in terms of the average clustering coefficient, the average characteristic path length, and the small-worldness, as well as their mean values, computed from the first three levels of both Type I and Type II of the reproducibility dataset.

**Table A1.** The mean and standard deviations of the seven network metrics of interest, for the three granularity levels in Type I and Type II, as computed across the 49 individual networks in each of the two tests of the reproducibility experiments, at a threshold of 0.3.

|  | Type I - Level 1 | | Type I - Level 2 | | Type I - Level 3 | |
| --- | --- | --- | --- | --- | --- | --- |
|  | test1 | test2 | test2 | test2 | test1 | test2 |
| Average Global Efficiency | 0.956±0.006 | 0.965±0.007 | 0.971±0.008 | 0.981±0.007 | 0.986±0.015 | 0.991±0.006 |
| Average Local Efficiency | 0.898±0.012 | 0.915±0.015 | 0.932±0.017 | 0.958±0.012 | 0.968±0.029 | 0.98±0.015 |
| Average Nodal Degree | 220.356±5.611 | 229.644±7.678 | 119.13±3.829 | 125.929±3.017 | 50.305±2.148 | 50.891±1.48 |
| Average Nodal Betweenness | 76.464±12.601 | 79.943±3.83 | 8.66±1.754 | 5.473±1.619 | 1.057±0.828 | 1.056±0.781 |
| Average Clustering Coefficient | 0.918±0.009 | 0.935±0.011 | 0.949±0.01 | 0.968±0.008 | 0.981±0.017 | 0.983±0.009 |
| Average Characteristic Path Length | 1.114±0.016 | 1.093±0.018 | 1.073±0.019 | 1.044±0.014 | 1.034±0.032 | 1.021±0.015 |
| Small-Worldness | 1.012±0.007 | 1.011±0.008 | 1.119±0.013 | 1.121±0.013 | 1.079±0.032 | 1.082±0.022 |
|  | Type II - Level 1 | | Type II - Level 2 | | Type II - Level 3 | |
|  | test1 | test2 | test1 | test2 | test1 | test2 |
| Average Global Efficiency | 0.962±0.007 | 0.964±0.008 | 0.984±0.013 | 0.988±0.006 | 0.991±0.012 | 0.993±0.006 |
| Average Local Efficiency | 0.911±0.016 | 0.917±0.017 | 0.963±0.027 | 0.974±0.013 | 0.978±0.025 | 0.984±0.014 |
| Average Nodal Degree | 162.813±5.817 | 165.473±6.108 | 64.476±2.899 | 65.663±1.553 | 49.17±1.745 | 49.37±1.326 |
| Average Nodal Betweenness | 26.639±13.96 | 28.836±12.148 | 1.988±1.219 | 1.573±0.787 | 0.698±0.66 | 0.795±0.662 |
| Average Clustering Coefficient | 0.929±0.012 | 0.935±0.013 | 0.976±0.015 | 0.979±0.009 | 0.987±0.014 | 0.987±0.008 |
| Average Characteristic Path Length | 1.098±0.019 | 1.09±0.02 | 1.04±0.031 | 1.027±0.014 | 1.023±0.028 | 1.017±0.014 |
| Small-Worldness | 1.155±0.227 | 1.141±0.185 | 1.072±0.023 | 1.072±0.018 | 1.075±0.037 | 1.079±0.032 |

**Table A2.** The mean and standard deviations of the test-retest differences, in terms of the seven network metrics of interest, for the three granularity levels in Type I and Type II, as computed across the 49 subjects involved in the reproducibility experiment at a threshold of 0.4.

|  | Type I - Level 1 | Type I - Level 2 | Type I - Level 3 |
| --- | --- | --- | --- |
| Average Global Efficiency | 0.016±0.012 | 0.02±0.013 | 0.02±0.017 |
| Average Local Efficiency | 0.032±0.022 | 0.042±0.029 | 0.035±0.026 |
| Average Nodal Degree | 0.066±0.04 | 0.076±0.047 | 0.042±0.028 |
| Average Nodal Betweenness | 0.142±0.395 | 0.082±0.4 | 0.042±0.286 |
| Average Clustering Coefficient | 0.022±0.014 | 0.008±0.008 | 0.013±0.011 |
| Average Characteristic Path Length | 0.038±0.026 | 0.004±0.013 | 0.01±0.035 |
| Small-Worldness | 0.012±0.009 | 0.006±0.004 | 0.015±0.012 |
|  | Type II - Level 1 | Type II - Level 2 | Type II - Level 3 |
| Average Global Efficiency | 0.014±0.012 | 0.0506±0.02 | 0.016±0.014 |
| Average Local Efficiency | 0.028±0.021 | 0.037±0.027 | 0.028±0.021 |
| Average Nodal Degree | 0.052±0.039 | 0.047±0.035 | 0.04±0.026 |
| Average Nodal Betweenness | 0.0816±0.39 | 0.082±0.4 | 0.001±0.002 |
| Average Clustering Coefficient | 0.044±0.037 | 0.01±0.009 | 0.013±0.014 |
| Average Characteristic Path Length | 0.032±0.025 | 0.007±0.027 | 0.01±0.037 |
| Small-Worldness | 0.139±0.1 | 0.009±0.008 | 0.015±0.011 |

**Table A3.** The ICC values of the seven network metrics of interest, for the three granularity levels in Type I and Type II, as computed across the 49 subjects involved in the reproducibility experiment at a threshold of 0.4.

|  | Type I - Level 1 | Type I - Level 2 | Type I - Level 3 |
| --- | --- | --- | --- |
| Average Global Efficiency | 0.918 | 0.964 | 0.776 |
| Average Local Efficiency | 0.943 | 0.975 | 0.756 |
| Average Nodal Degree | 0.963 | 0.981 | 0.443 |
| Average Nodal Betweenness | 0.71 | 0.973 | 0.892 |
| Average Clustering Coefficient | 0.95 | 0.98 | 0.587 |
| Average Characteristic Path Length | 0.942 | 0.971 | 0.809 |
| Small-Worldness | 0.951 | 0.954 | 0.989 |
|  | Type II - Level 1 | Type II - Level 2 | Type II - Level 3 |
| Average Global Efficiency | 0.667 | 0.817 | 0.79 |
| Average Local Efficiency | 0.593 | 0.801 | 0.803 |
| Average Nodal Degree | 0.707 | 0.855 | 0.989 |
| Average Nodal Betweenness | 0.71 | 0.872 | 0.816 |
| Average Clustering Coefficient | 0.592 | 0.664 | 0.91 |
| Average Characteristic Path Length | 0.578 | 0.822 | 0.506 |
| Small-Worldness | 0.934 | 0.772 | 0.912 |

**Table A4.** The mean and standard deviations of the test-retest differences, in terms of the seven network metrics of interest, for the three granularity levels in Type I and Type II, as computed across the 49 subjects involved in the reproducibility experiment at a threshold of 0.5.

|  | Type I - Level 1 | Type I - Level 2 | Type I - Level 3 |
| --- | --- | --- | --- |
| Average Global Efficiency | 0.023±0.015 | 0.034±0.024 | 0.024±0.019 |
| Average Local Efficiency | 0.048±0.031 | 0.062±0.041 | 0.042±0.03 |
| Average Nodal Degree | 0.08±0.052 | 0.096±0.053 | 0.047±0.034 |
| Average Nodal Betweenness | 0.16±0.41 | 0.082±0.4 | 0.041±0.286 |
| Average Clustering Coefficient | 0.024±0.016 | 0.006±0.006 | 0.011±0.011 |
| Average Characteristic Path Length | 0.062±0.041 | 0.003±0.012 | 0.009±0.031 |
| Small-Worldness | 0.017±0.012 | 0.003±0.002 | 0.007±0.005 |
|  | Type II - Level 1 | Type II - Level 2 | Type II - Level 3 |
| Average Global Efficiency | 0.02±0.019 | 0.0357±0.02 | 0.02±0.015 |
| Average Local Efficiency | 0.041±0.032 | 0.046±0.034 | 0.035±0.029 |
| Average Nodal Degree | 0.063±0.039 | 0.048±0.041 | 0.046±0.032 |
| Average Nodal Betweenness | 0.0816±0.39 | 0.082±0.4 | 0±0 |
| Average Clustering Coefficient | 0.063±0.046 | 0.007±0.008 | 0.012±0.013 |
| Average Characteristic Path Length | 0.053±0.041 | 0.007±0.024 | 0.009±0.033 |
| Small-Worldness | 0.086±0.061 | 0.004±0.003 | 0.01±0.006 |

**Table A5.** The ICC values of the seven network metrics of interest, for the three granularity levels in Type I and Type II, as computed across the 49 subjects involved in the reproducibility experiment at a threshold of 0.5.

|  | Type I - Level 1 | Type I - Level 2 | Type I - Level 3 |
| --- | --- | --- | --- |
| Average Global Efficiency | 0.959 | 0.97 | 0.827 |
| Average Local Efficiency | 0.949 | 0.98 | 0.557 |
| Average Nodal Degree | 0.958 | 0.983 | 0.944 |
| Average Nodal Betweenness | 0.601 | 0.967 | 0.899 |
| Average Clustering Coefficient | 0.95 | 0.97 | 0.764 |
| Average Characteristic Path Length | 0.947 | 0.975 | 0.636 |
| Small-Worldness | 0.922 | 0.934 | 0.924 |
|  | Type II - Level 1 | Type II - Level 2 | Type II - Level 3 |
| Average Global Efficiency | 0.748 | 0.797 | 0.729 |
| Average Local Efficiency | 0.663 | 0.646 | 0.774 |
| Average Nodal Degree | 0.504 | 0.644 | 0.873 |
| Average Nodal Betweenness | 0.79 | 0.644 | 0.82 |
| Average Clustering Coefficient | 0.81 | 0.583 | 0.62 |
| Average Characteristic Path Length | 0.642 | 0.69 | 0.546 |
| Small-Worldness | 0.889 | 0.55 | 0.903 |

**Table A6.** The mean and standard deviations of the test-retest differences, in terms of the seven network metrics of interest, for the three granularity levels in Type I and Type II, as computed across the 49 subjects involved in the reproducibility experiment at a threshold of 0.6.

|  | Type I - Level 1 | Type I - Level 2 | Type l - Level 3 |
| --- | --- | --- | --- |
| Average Global Efficiency | 0.038±0.022 | 0.043±0.026 | 0.027±0.019 |
| Average Local Efficiency | 0.064±0.042 | 0.085±0.05 | 0.051±0.039 |
| Average Nodal Degree | 0.093±0.066 | 0.115±0.061 | 0.055±0.046 |
| Average Nodal Betweenness | 0.161±0.296 | 0.082±0.4 | 0.041±0.286 |
| Average Clustering Coefficient | 0.031±0.021 | 0.004±0.005 | 0.009±0.012 |
| Average Characteristic Path Length | 0.097±0.064 | 0.003±0.01 | 0.008±0.028 |
| Small-Worldness | 0.021±0.015 | 0.001±0.001 | 0.004±0.003 |
|  | Type II - Level 1 | Type II - Level 2 | Type II - Level 3 |
| Average Global Efficiency | 0.027±0.024 | 0.0285±0.01 | 0.026±0.019 |
| Average Local Efficiency | 0.049±0.039 | 0.052±0.045 | 0.044±0.035 |
| Average Nodal Degree | 0.069±0.054 | 0.052±0.054 | 0.053±0.041 |
| Average Nodal Betweenness | 0.0816±0.39 | 0.082±0.4 | 0±0 |
| Average Clustering Coefficient | 0.07±0.05 | 0.006±0.009 | 0.008±0.01 |
| Average Characteristic Path Length | 0.072±0.056 | 0.006±0.021 | 0.008±0.029 |
| Small-Worldness | 0.043±0.034 | 0.002±0.002 | 0.005±0.003 |

**Table A7.** The ICC values of the seven network metrics of interest, for the three granularity levels in Type I and Type II, as computed across the 49 subjects involved in the reproducibility experiment at a threshold of 0.6.

|  | Type I - Level 1 | Type I - Level 2 | Type I - Level 3 |
| --- | --- | --- | --- |
| Average Global Efficiency | 0.978 | 0.982 | 0.631 |
| Average Local Efficiency | 0.965 | 0.982 | 0.67 |
| Average Nodal Degree | 0.935 | 0.978 | 0.518 |
| Average Nodal Betweenness | 0.819 | 0.859 | 0.856 |
| Average Clustering Coefficient | 0.97 | 0.976 | 0.634 |
| Average Characteristic Path Length | 0.93 | 0.978 | 0.526 |
| Small-Worldness | 0.894 | 0.826 | 0.892 |
|  | Type II - Level 1 | Type II - Level 2 | Type II - Level 3 |
| Average Global Efficiency | 0.872 | 0.717 | 0.711 |
| Average Local Efficiency | 0.736 | 0.652 | 0.774 |
| Average Nodal Degree | 0.974 | 0.731 | 0.861 |
| Average Nodal Betweenness | 0.627 | 0.945 | 0.874 |
| Average Clustering Coefficient | 0.594 | 0.451 | 0.92 |
| Average Characteristic Path Length | 0.753 | 0.729 | 0.601 |
| Small-Worldness | 0.704 | 0.74 | 0.524 |

**Table A8.** The mean and standard deviations of the test-retest differences, in terms of the seven network metrics of interest, for the three granularity levels in Type I and Type II, as computed across the 49 subjects involved in the reproducibility experiment at a threshold of 0.7.

|  | Type I - Level 1 | Type I - Level 2 | Type I - Level 3 |
| --- | --- | --- | --- |
| Average Global Efficiency | 0.046±0.036 | 0.053±0.037 | 0.031±0.023 |
| Average Local Efficiency | 0.086±0.062 | 0.107±0.067 | 0.056±0.044 |
| Average Nodal Degree | 0.099±0.084 | 0.114±0.079 | 0.07±0.059 |
| Average Nodal Betweenness | 0.178±0.309 | 0.082±0.4 | 0.041±0.286 |
| Average Clustering Coefficient | 0.033±0.027 | 0.003±0.004 | 0.007±0.011 |
| Average Characteristic Path Length | 0.164±0.121 | 0.003±0.009 | 0.007±0.025 |
| Small-Worldness | 0.02±0.014 | 0.001±0 | 0.002±0.001 |
|  | Type II - Level 1 | Type II - Level 2 | Type II - Level 3 |
| Average Global Efficiency | 0.036±0.028 | 0.0194±0.01 | 0.032±0.024 |
| Average Local Efficiency | 0.063±0.055 | 0.058±0.052 | 0.056±0.041 |
| Average Nodal Degree | 0.092±0.08 | 0.077±0.071 | 0.065±0.059 |
| Average Nodal Betweenness | 0.0816±0.39 | 0.082±0.4 | 0±0.001 |
| Average Clustering Coefficient | 0.061±0.044 | 0.004±0.008 | 0.006±0.01 |
| Average Characteristic Path Length | 0.117±0.105 | 0.005±0.019 | 0.007±0.025 |
| Small-Worldness | 0.019±0.017 | 0.001±0.001 | 0.002±0.002 |

**Table A9.** The ICC values of the seven network metrics of interest, for the three granularity levels in Type I and Type II, as computed across the 49 subjects involved in the reproducibility experiment at a threshold of 0.7.

|  | Type I - Level 1 | Type I - Level 2 | Type I - Level 3 |
| --- | --- | --- | --- |
| Average Global Efficiency | 0.942 | 0.976 | 0.598 |
| Average Local Efficiency | 0.952 | 0.977 | 0.57 |
| Average Nodal Degree | 0.875 | 0.949 | 0.783 |
| Average Nodal Betweenness | 0.923 | 0.818 | 0.761 |
| Average Clustering Coefficient | 0.922 | 0.965 | 0.949 |
| Average Characteristic Path Length | 0.945 | 0.973 | 0.168 |
| Small-Worldness | 0.992 | 0.695 | 0.944 |
|  | Type II - Level 1 | Type II - Level 2 | Type II - Level 3 |
| Average Global Efficiency | 0.62 | 0.72 | 0.667 |
| Average Local Efficiency | 0.352 | 0.961 | 0.774 |
| Average Nodal Degree | 0.766 | 0.827 | 0.992 |
| Average Nodal Betweenness | 0.362 | 0.933 | 0.812 |
| Average Clustering Coefficient | 0.694 | 0.523 | 0.864 |
| Average Characteristic Path Length | 0.767 | 0.992 | 0.914 |
| Small-Worldness | 0.808 | 0.932 | 0.864 |
